# Metabolic Syndrome Features: Is There a Modulation Role by Mineral Water Consumption? A Review

**DOI:** 10.3390/nu11051141

**Published:** 2019-05-22

**Authors:** Daniela Costa-Vieira, Rosário Monteiro, Maria João Martins

**Affiliations:** 1 Departamento de Biomedicina, Unidade de Bioquímica, Faculdade de Medicina, Universidade do Porto, 4200-319 Porto, Portugal; mimed1301215@med.up.pt (D.C.-V.); rosariom@med.up.pt (R.M.); 2 I3S—Instituto de Investigação e Inovação em Saúde, Universidade do Porto, 4200-135 Porto, Portugal; 3 Administração Regional de Saúde do Norte, 4000-477 Porto, Portugal

**Keywords:** blood pressure, dyslipidemia, glucose, metabolic syndrome, mineral water, obesity

## Abstract

Metabolic syndrome (MetSyn) promotes, among others, the development of atherosclerotic cardiovascular disease and diabetes. Its prevalence increases with age, highlighting the relevance of promoting precocious MetSyn primary prevention and treatment with easy-to-implement lifestyle interventions. MetSyn features modulation through mineral water consumption was reviewed on Pubmed, Scopus and Google Scholar databases, using the following keywords: metabolic syndrome, hypertension, blood pressure (BP), cholesterol, triglycerides, apolipoprotein, chylomicron, very low-density lipoprotein, low-density lipoprotein, high-density lipoprotein (HDL), glucose, insulin, body weight, body mass index, waist circumference (WC), obesity and mineral(-rich) water. Twenty studies were selected: 12 evaluated BP, 13 assessed total-triglycerides and/or HDL-cholesterol, 10 analysed glucose and/or 3 measured WC. Mineral waters were tested in diverse protocols regarding type and composition of water, amount consumed, diet and type and duration of the study. Human and animal studies were performed in populations with different sizes and characteristics. Distinct sets of five studies showed beneficial effects upon BP, total-triglycerides, HDL-cholesterol and glucose. WC modulation was not reported. Minerals/elements and active ions/molecules present in mineral waters (and their pH) are crucial to counterbalance their inadequate intake and body status as well as metabolic dysfunction and increased diet-induced acid-load observed in MetSyn. Study characteristics and molecular/physiologic mechanisms that could explain the different effects observed are discussed. Further studies are warranted for determining the mechanisms involved in the putative protective action of mineral water consumption against MetSyn features.

## 1. Introduction 

The metabolic syndrome (MetSyn) represents a cluster of multiple and interrelated metabolic features: high blood pressure (BP), dyslipidemia [raised fasting total-triglycerides and lowered fasting high-density lipoprotein (HDL) cholesterol], raised fasting glycemia and obesity (linked to an excess of visceral abdominal fat). These features promote the development of non-alcoholic fatty liver disease, type 2 diabetes mellitus (T2DM), atherosclerotic cardiovascular disease and cancer, which are prime causes of morbidity and mortality worldwide. Although the etiology of MetSyn is still not entirely clear, it is known to involve complex interactions between genetic, metabolic and environmental factors [1,2,3,4,5,6,7,8,9]. Insulin resistance, altered redox state, endoplasmic reticulum stress, low grade pro-inflammatory state, hypercoagulable/prothrombotic state and endothelial dysfunction are also characteristics of the MetSyn [5,9,10,11,12,13,14,15].

The prevalence of MetSyn depends not only on the composition (in terms of age, gender, race and ethnicity), region and urban or rural environment of the population considered but also on the criteria used for its definition [3,5,16]. In 2006, the International Diabetes Federation (IDF) estimated that 20–25% of the world’s population had MetSyn [16]. The presence of one MetSyn feature increases the risk of developing MetSyn later in life and MetSyn prevalence increases with age [3]. When reviewing data regarding young adults, Nolan et al. found that MetSyn was present in 4.8–7% of individuals aged 18–30 years. Hence, precocious promotion of MetSyn primary prevention is most relevant for reducing the risk for and incidence of the aforementioned pathological conditions later in life [3].

In MetSyn management, diet assumes a central importance as there is no effective preventive approach beyond lifestyle-based interventions aimed at normalizing body weight and achieving and maintaining cardio-metabolic control [5,6,7]. Dietary approaches to stop hypertension (DASH) and Mediterranean diet have been reported to be essential both in the prevention and in the treatment of MetSyn and its individual features. Owing to their composition, among other factors, they share high mineral/element content and low diet-induced acid-load capacity [5,6,7,17,18]. The higher diet-induced acid-load capacity and lower mineral/element content that is typical of Western diets, along with impaired mineral/element status in the body, associate with MetSyn features and MetSyn itself as well as some MetSyn-related diseases [6,8,9,18,19,20,21,22,23,24,25,26,27,28,29].

Curiously, in 2009, the World Health Organization (WHO) highlighted the public health significance of water composition [30]. The consumption of water can make important contributions to mineral/element nutritional needs depending on its composition, and the need to promote the consumption of highly-mineralized water has been supported [30,31,32,33,34,35,36,37,38,39,40,41,42,43,44,45,46,47]. In this regard, as examples, it has been disclosed that waters containing 60 mg magnesium/L can provide between 30% and 102% [31] and those containing 100–150 mg calcium/L can provide 10–31% [32] of magnesium and calcium recommended dietary allowances, respectively, depending on the amount of water consumed (that relates to the age of the individual). So, in a MetSyn/obesity setting, mineral(-rich) waters could provide significant amounts of energy-free minerals/elements [36,41,42,43,44,47]. In fact, minerals/elements from water are well-absorbed and highly bioavailable [30,31,32,33,34,35,36,37,38,39,40,41,42,43,44,45,47,48,49] and because of their chemical presentation forms in water they could be more readily and easily absorbed, and so more bioavailable, from water than from food [30,34,44,47,48]. Accordingly, calcium from water has been reported to present equal [30,32,33,34,36,39,43,45,47] or even higher [33,34,36,39,40,45,47] absorption and/or bioavailability than from milk or pharmaceutical supplements. Similar findings were reported for magnesium from water versus food or magnesium pharmacological preparations [30,34,38,41,44,48]. In addition to water mineral/element content, and among the parameters taken into consideration for classification of mineral waters, pH and bicarbonate and hydrogen sulphide content are also quite relevant to human hydrosaline balance and metabolic health ([9,40,46,47,50,51,52,53] further discussed below).

Natural mineral waters are originated from underground sources, which allow their physicochemical composition and organoleptic characteristics to remain practically constant and intact while protecting them from any risk of contamination. Natural mineral waters possess health promoting properties [40,46,47,54,55].

Hence, we aimed to review published data on the effects of mineral water consumption on the metabolic features included in the MetSyn definition [1]: BP and fasting total-triglycerides, HDL-cholesterol and glucose as well as waist circumference (WC).

## 2. Methods

The present literature review was performed on Pubmed, Scopus and Google Scholar databases, up to 18 January 2019. The following keywords were used for the search of the effects of mineral water consumption upon MetSyn features: metabolic syndrome, hypertension, BP, cholesterol, triglycerides, apolipoprotein, chylomicron, very low-density lipoprotein (VLDL), low-density lipoprotein (LDL), HDL, glucose, insulin, body weight, body mass index, WC, obesity and mineral(-rich) water. The articles chosen to be discussed in this review were selected in a 3-step process: (a) aiming the first selection phase, the titles of all searches found were read, (b) secondly, the abstracts of the articles elected previously were read and (c) finally, the articles picked in (b) were read. In the context of mineral(-rich) water consumption and MetSyn/MetSyn features, we selected only full experimental articles, including studies in humans (without age, sex or race restrictions) and in animal models, randomized and non-randomized interventions, blinded and non-blinded interventions, cross-over, sequential and parallel trials, acute and chronic studies (the latter with variable durations) (Table 1, Table 2, Table 3 and Table 4).

## 3. Results

Mineral waters were tested in diverse protocols in terms of type and composition of water, amount consumed, diet and type and duration of the study. Human studies were performed in populations with different sizes and characteristics; the same for rat strains in the animal studies.

Amid the 20 studies selected (a) 12 evaluated BP (Table 1), (b) 13 assessed the lipid profile [we considered 3 studies regarding the impact upon the postprandial lipid profile aiming to increase the strength of the discussion as metabolism of postprandial lipoproteins and metabolism of fasting lipoproteins are closely associated (in 2 out of these 3 studies both nutritional states were evaluated); Table 2], (c) 10 analysed glucose [we included 4 studies regarding the impact upon the postprandial glucose homeostasis also aiming to increase the strength of the discussion (in 2 out of these 4 studies both nutritional states were evaluated); Table 3] and/or (d) 3 studies measured WC (owing to this low number, we also considered here the studies that appraised body weight and/or body mass index without WC, n = 6; Table 4).

### 3.1. Metabolic Syndrome Features Modulation by Mineral Water Consumption—Blood Pressure

Among the 12 studies selected: 1 was carried out in a rodent model and 11 in humans (Table 1).

BP was evaluated alone or together with several BP-related parameters, in distinct combinations: resting and/or ambulatory systolic, diastolic and mean BP [49,56,57,58,59,60,61,62,63,64,65,66], resting and/or ambulatory heart rate [49,56,57,64], circulating minerals/electrolytes [49,56,57,59,65], renin activity [56,62], atrial natriuretic factor [62], aldosterone [56,57,62,63] and catecholamines [56,62], circulating [56,57,62] and urinary [56] uric acid (and circulating urea [57]) and glomerular filtration rate [62] as well as urinary pH [58,63,64], acid-base status [62] and urea [60] and mineral/electrolyte [49,56,57,58,59,60,62,63,64] excretion (Table 1).

Five studies described a beneficial effect of mineral water intake on BP [56,57,58,59,60]: two measured heart rate [56,57], two evaluated the hormonal and enzymatic regulation of BP [56,57], all of them measured circulating [56,57,59] and/or urine [56,57,58,59,60] minerals/electrolytes (namely sodium, chloride, bicarbonate, calcium, magnesium, potassium and/or phosphorous, phosphate), two measured circulating uric acid (and one quantified circulating urea [57]) [56,57] and three directly or indirectly assessed urine acid-base status (urinary pH, uric acid or urea) [56,58,60,67]. BP reduction (positive result) was obtained with the following mineral waters: bicarbonate- and sodium-rich, mineral-rich water [57], bicarbonate- and sodium-rich as well as magnesiac, mineral-rich water [56], bicarbonate-, sodium- and chloride-rich, mineral-rich water [58], sulphate-rich, calcic and magnesiac, mineral-rich water [59] and magnesiac, mineral water [60] (Table 1).

Six studies found no effect of mineral waters intake upon BP [49,61,63,64,65,66], while analyzing very few BP-related parameters: two assessed heart rate [49,64], one evaluated the hormonal regulation of BP [63], two measured urinary pH [63,64], three assessed minerals/electrolytes in urine [49,63,64] and two in circulation [49,65]. In one study, the decrease in BP induced by a low-sodium diet was avoided by mineral-rich water ingestion [62]. This set of studies tested the following mineral waters: bicarbonate-, sodium- and chloride-rich as well as fluorurate, mineral-rich water [61], bicarbonate-, sodium- and chloride-rich, mineral-rich water [63,66], bicarbonate- and sodium-rich, mineral-rich water [64], bicarbonate-, sodium-, chloride- and sulphate-rich, mineral-rich water [62], bicarbonate- and sodium-rich as well as magnesiac, mineral-rich water [62], bicarbonate-, sodium-, chloride- and sulphate-rich as well as magnesiac, mineral-rich water [65] and magnesiac, mineral water [49] (Table 1).

### 3.2. Metabolic Syndrome Features Modulation by Mineral Water Consumption—Lipid Profile

Among the 13 studies selected (3 described the impact upon the postprandial circulating lipid profile [66,68,69]): 2 were carried out in rodent models and 11 in humans (Table 2). Several features of the lipid profile as well as lipid profile-related parameters were evaluated in distinct combinations: circulating total-, VLDL- and chylomicron-triglycerides [57,58,61,62,63,65,66,68,69,70,71,72,73], circulating total-, chylomicron-, VLDL-, LDL- and HDL-cholesterol [57,58,61,62,63,65,66,68,70,71,72,73], circulating oxidized LDL [63], circulating lipoprotein(a) [65], circulating apolipoproteins (Apo) AI and B [58,61,63,65], HDL-cholesterol/ApoAI, LDL-cholesterol/ApoB, HDL-cholesterol/total-cholesterol (or total-cholesterol/HDL-cholesterol), total-cholesterol/LDL-cholesterol and HDL-cholesterol/LDL-cholesterol (or LDL-cholesterol/HDL-cholesterol) ratios [57,58,61,63,65,70], fecal [65,70] and circulating [73] total bile acids, intestinal propulsion [70] and bowel movements [73], biliary elimination curve and total bile acids and cholesterol in bile [70], gallbladder volume and ejection fraction [65,69,73] and circulating cholecystokinin [69] and cortisol [71] (Table 2).

Regarding the effects of mineral-rich water ingestion upon lipid profile and/or lipid profile-related parameters, no negative effects were found while 3 studies revealed no effect [62,71,73], 3 studies revealed no difference in the effect between the control and test waters [63,66,72], 2 studies presented no effect upon the lipid parameters included in MetSyn definition [1] but showed other positive effects [65,70] and 5 studies showed positive effects upon the lipid parameters included in MetSyn definition [regardless of the nutritional state (as explained above) as well as upon others [57,58,61,68,69]] (Table 2).

The following mineral-rich waters were tested: bicarbonate- and sodium-rich [57], bicarbonate-, sodium- and chloride-rich [58,63,66,69], bicarbonate-, sodium- and chloride-rich as well as fluorurate [61,68], bicarbonate- and sodium-rich as well as magnesiac [62], bicarbonate-, sodium-, chloride- and sulphate-rich [62], bicarbonate-, sodium-, chloride- and sulphate-rich as well as magnesiac [65], bicarbonate-, sodium-, chloride- and sulphate-rich as well as sulphurous, calcic, magnesiac and fluorurate [70], bicarbonate-, sodium- and sulphate-rich as well as magnesiac and calcic [71], bicarbonate-rich and calcic [72] and bicarbonate- and sulphate-rich as well as magnesiac, calcic and fluorurate [73] (Table 2).

### 3.3. Metabolic Syndrome Features Modulation by Mineral Water Consumption—Glucose

Ten studies were selected (including 4 regarding the impact upon the postprandial circulating glucose [62,66,69,75]): 2 carried out in rodent models and 8 in humans, which can be seen in Table 3. Several features regarding glucose homeostasis were evaluated in distinct combinations: circulating glucose [57,58,61,62,63,66,69,71,74,75], glycoalbumin [71], glycated hemoglobin [74], insulin [57,58,62,63,69,71,74,75], C-peptide [74], leptin [57] and insulin-growth factor-1 [74], insulin sensitivity index [57], homeostasis model assessment index/ratio (HOMA/HOMAR) [71,75] and quantitative insulin sensitivity check index (QUICKI) [75] (Table 3). Regarding the effects of mineral-rich water ingestion upon glucose and/or glucose homeostasis-related parameters, no undesirable effects were found while 2 studies revealed no effect [62,66], 1 study revealed no difference in the effect between the control and test waters [63], 2 studies presented no effect upon glucose but showed other advantageous effects [69,75] and 5 studies showed beneficial effects upon glucose (as well as upon other parameters) [57,58,61,71,74] (Table 3).

The following mineral-rich waters were tested: bicarbonate- and sodium-rich [57], bicarbonate-, sodium- and chloride-rich as well as fluorurate [61,75], bicarbonate-, sodium- and chloride-rich [58,63,66,69], bicarbonate- and sodium-rich as well as magnesiac [62], bicarbonate-, sodium-, chloride- and sulphate-rich [62], bicarbonate-, sodium- and sulphate-rich as well as magnesiac and calcic [71] and chloride-, sodium- and sulphate-rich as well as sulphurous, calcic and fluorurate [74] (Table 3).

### 3.4. Metabolic Syndrome Features Modulation by Mineral Water Consumption—Waist Circumference

Nine studies were selected: 3 carried out in rodent models and 6 in humans, which can be seen in Table 4. WC was evaluated along with other obesity-related measures such as body weight and body mass index [58,61,63] that in turn were also assessed alone (body weight) or together [56,57,64,70,73,74]. No modulation of WC and body mass index nor a negative impact upon body weight was found. The prevention of treatment-induced body weight loss [70,74], in addition to the maintenance of body weight despite increased food intake [73], was observed as a consequence of mineral-rich water ingestion.

The following mineral-rich waters were tested: bicarbonate- and sodium-rich as well as magnesiac [56], bicarbonate- and sodium-rich [57,64], bicarbonate-, sodium- and chloride-rich [58,63], bicarbonate-, sodium- and chloride-rich as well as fluorurate [61], chloride-, sodium- and sulphate-rich as well as sulphurous, calcic and fluorurate [74], bicarbonate-, sodium-, chloride- and sulphate-rich as well as sulphurous, calcic, magnesiac and fluorurate [70] and bicarbonate- and sulphate-rich as well as magnesiac, calcic and fluorurate [73] (Table 4).

## 4. Discussion

### 4.1. Metabolic Syndrome Features—Blood Pressure

High BP defined by systolic BP (SBP) ≥ 130 mmHg and/or diastolic BP (DBP) ≥ 85 mmHg is one of the features considered for MetSyn clinical diagnosis; antihypertensive drug treatment in a patient with hypertension history can be used as an alternate indicator [1]. Hypertension is a global public health issue that leads to premature death and disability, also being a major risk factor for cardiovascular and renal diseases as well as retinal hemorrhage and visual impairment. According to the WHO, ischemic heart disease and stroke were the 2 most important global causes of death in 2016, highlighting hypertension as an important preventable cause of death [76,77,78,79,80,81,82]. Worldwide, the number of adults with raised BP (SBP ≥ 140 mmHg or DBP ≥ 90 mmHg) increased from 594 million in 1975 to 1.13 billion in 2015, being 24.1% in men and 20.1% in women [82].

#### Metabolic Syndrome Features Modulation by Mineral Water Consumption—Blood Pressure

Although among the 12 studies selected we had noticed no report of increased BP upon mineral water ingestion, Schorr et al. found that the ingestion of a NaCl-rich mineral-rich water (also bicarbonate- and sulphate-rich) abolished the mean resting BP significant (and expected [83]) reduction induced by the consumption of a sodium-restricted diet + low-sodium mineral water in elderly healthy normotensives, progressively from the 1st to the 4th week of treatment. In this sodium-restricted population, the consumption of a NaHCO_3_^−^-rich mineral-rich water (also magnesiac) allowed a significant reduction of mean resting BP similar to the decrease found with low-sodium mineral water, at both time points. With these two waters, urinary calcium excretion was significantly reduced at week 4, improving calcium homeostasis. Nevertheless, it should be stated that sodium concentration was approximately 2 times higher in NaCl-rich than in NaHCO_3_^−^-rich mineral-rich water [62].

Also associated with a salt-restricted diet, Luft et al. described a significant decrease of SBP in mildly hypertensives but not in normotensives (the former group presenting BP 15 mmHg higher than the latter) as a consequence of mineral-rich water (bicarbonate- and sodium-rich as well as magnesiac) intake, for 7 days. No influence upon BP in both BP groups by NaCl-containing control water + low-sodium diet was observed. Concomitantly, a significant modulation of fasting circulating electrolytes consistent with a putative plasma volume expansion was observed: increase of chloride and decrease of bicarbonate in both BP groups (non-significantly in hypertensives for the latter ion) with NaCl-containing control water versus NaHCO_3_^−^-containing mineral-rich water (within a low-sodium diet setting). Also, urinary calcium and chloride excretion was significantly increased with NaCl- but not with NaHCO_3_^−^-containing water [56]. Among the 12 selected studies on the effect of mineral water consumption upon BP, only the 2 discussed above compared sodium-rich waters containing different sodium salts [56,62]. Excessive cellular electrolyte redistribution and/or intracellular sodium or chloride accumulation, extracellular fluid volume enlargement, plasma volume expansion and lower urinary sodium excretion with NaCl versus NaHCO_3_^−^ explain their divergent behaviour towards BP. In addition, chloride itself may act as a direct vasoconstrictor [56,58,84,85,86]. In the 2 aforementioned interventions, the deviating effect observed for NaCl-containing waters upon BP of normotensives most probably resulted from the different duration of the treatment and age of the individuals, as a similar total daily amount of sodium was ingested (the same for the NaCl quantity provided by the amount of water drank). The NaHCO_3_^−^-containing mineral waters tested (with quite similar compositions but in different amounts) showed an absence of effect upon BP on normotensive subjects [56,62].

In addition to the reports from Luft et al. [56], both Pereira et al. [57] and Pérez-Granados et al. [58] reported beneficial effects of distinct mineral-rich waters (NaHCO_3_^−^-rich) upon BP on a metabolic dysfunction background. In dietary interventions that lasted 8 weeks, Pereira et al. found a protection against detrimental fructose effects upon SBP and DBP in Sprague–Dawley rats in the initial weeks of the experimental protocol [57] and Pérez-Granados et al. described a small significant SBP decrease in moderately hypercholesterolemic young subjects (here, water was also chloride-rich) [58]. Erectile dysfunction associates with endothelial dysfunction, raised BP and systemic cardiovascular disease [87,88,89]. Remarkably, Pereira et al. showed that, in the aforesaid rodent model, the intake of the bicarbonate- and sodium-rich mineral-rich water markedly tended to increase cavernous Sirt1, a protein with documented protective roles in the vascular system [87].

Urinary sodium and/or chloride excretion mirrored sodium and chloride ingestion among the aforesaid studies [56,57,58,62]. Remarkably, both Schorr et al. [62] and Luft et al. [56] testified a differential protective effect upon urinary calcium excretion of NaHCO_3_^−^- versus NaCl-containing water comparable to BP modulation. Moreover, curiously and oppositely, a beneficial modulation of BP has been brought up by Rylander et al. in two distinct populations presenting low urinary calcium and/or magnesium excretion and hence presenting some level of deficiency in these ions [59,60]. Firstly, Rylander et al. reported a significant decrease of SBP and DBP concomitant with a significant increase of urinary magnesium excretion, but not calcium, after mineral-rich water (rich in sulphate, calcium and magnesium but not in bicarbonate) intake by individuals with borderline hypertension and low urinary magnesium and calcium excretion, for 4 weeks. An absence of effect upon circulating electrolytes/minerals was noticed [59]. Then, Rylander et al. showed a tendency towards an increase of urinary magnesium and calcium excretion after consumption of mineral water (magnesiac, not containing bicarbonate) by healthy subjects, for 2 weeks, associated with a tendency to decrease DBP within the sub-set of individuals with low and high urinary magnesium and urea excretion, respectively [60]. Additionally, this group of researchers confirmed a direct positive significant association between urine acidity (measured as urinary urea excretion) and urinary calcium, magnesium and potassium excretion as well as a significant relation between lower urinary magnesium excretion and higher SBP among those with high urinary acidity, hence revealing some level of impaired magnesium body status [60]. In this regard, an inverse relationship between dietary magnesium intake and the risk of hypertension has been reported [26] and similar findings were reported for potassium and calcium [18,29]. Interestingly, relevant to BP regulation, the ingestion of mineral waters might contribute to mineral homeostasis by increasing their intake and, consequently, increasing urinary mineral excretion and/or by reducing urine acidity and, consequently, decreasing urinary mineral excretion. Regarding the latter topic, the bicarbonate content of mineral water has a prime role in the control of urinary acidity since it contributes to increased urinary pH [50,52,90,91,92,93,94]. However, with NaHCO_3_^−^-rich mineral-rich waters, although Pérez-Granados et al. [58] described an increase of urinary pH (in this trial, water was also chloride-rich), Luft et al. [56] reported no difference for urinary uric acid excretion, an indirect marker of urine pH [67], as well as for circulating uric acid (here, water was also magnesiac); nevertheless, as already stated, both research groups found beneficial effects upon BP. Interestingly, some epidemiological studies suggested the existence of an association between elevated circulating uric acid and cardiovascular diseases, including hypertension [18,67,95,96]. The short duration of Luft et al. intervention might have hampered an effect upon circulating uric acid levels [56]. Schorr et al., also with NaHCO_3_^−^-rich and magnesiac mineral-rich water, did not find an overall mechanistic coincidence in modulation of BP and urinary calcium and net acid excretion results [62].

Bicarbonate, magnesium and calcium, among others, were present in relevant amounts [47] in the aforementioned mineral waters that beneficially modulated BP [56,57,58,59,60]. Diet-induced acid load associates with higher SBP, DBP and higher hypertension prevalence. Increased glucocorticoid secretion is needed to facilitate renal elimination of excess H^+^, elevated circulating cortisol frequently relates to hypertension and reduction of diet-induced acid load by administration of alkali salts (including bicarbonate) reduces glucocorticoid secretion [18,24,92]. In line, Pereira et al. disclosed a trend towards a reduction of fructose-induced fasting circulating corticosterone after the ingestion of bicarbonate- and sodium-rich mineral-rich water, for 8 weeks (in this protocol, a short fasting period was performed) [97]. Magnesium is a natural calcium channel blocker, ameliorates endothelial dysfunction, increases nitric oxide and induces direct and indirect vasodilation and vascular smooth muscle relaxation, which leads to the decrease of BP [9,91,98,99]. Magnesium also modulates the activity of the sympathetic nervous system, which contributes to BP and heart rate [9]. Calcium-rich diets suppress parathormone (PTH) and, subsequently, decrease vasoconstriction and BP [100]. PTH directly stimulate aldosterone synthesis [101].

However, with an increase of urinary pH and a decrease of urinary calcium excretion, Toxqui et al. [63], unlike Pérez-Granados et al. [58], found a lack of effect upon BP when testing a quite similar mineral-rich water (bicarbonate-, sodium- and chloride-rich) in the same amount also for 8 weeks, in a similar moderately hypercholesterolemic young population. Differences in population size, study design and urinary sodium excretion might justify the discrepancy found. Considering that the decrease of urinary calcium and potassium excretion (the latter also happened with the control water tested) contributed to improve their homeostasis, a beneficial BP modulation would have been expected [9,18,29,63,102]. Similarly, in moderately hypercholesterolemic men, Zair et al. found no effect upon BP with the intake of a strongly bicarbonated as well as chloride- and sodium-rich mineral-rich water for 8 weeks, both at basal and postprandial states (no BP-related parameters were explored) [66]. Additionally, in an intervention identical to the one described by Pérez-Granados et al. [58], and in which the measurement of BP-related parameters did not occur, Schoppen et al. observed no effects upon BP in healthy postmenopausal women, consuming a low-sodium diet [61]. An expected protective effect upon BP of a low-sodium intake [83] might have ruled out the putative beneficial impact of the mineral-rich water tested by Schoppen et al., as this mineral-rich water [61] was quite similar to the one tested by Pérez-Granados et al. [58]: the main difference being the fluoride concentration [58,61]. Again, with a NaHCO_3_^−^-rich mineral-rich water, an absence of effect upon BP, along with no change in urine pH and urinary electrolytes, was disclosed by Santos et al. in healthy normotensive volunteers in an intervention that lasted 7 weeks [64]. A very low chloride to sodium ratio in the mineral-rich water can clarify the absence of impact upon urinary sodium excretion observed by Santos et al. as the intestinal transport of sodium roughly parallels that of chloride [64] but in the case of Toxqui et al. such a very low ratio was not present [63]. Once more, in an older moderately hypercholesterolemic population, Capurso et al. found no effect upon BP with the intake of a mineral-rich water (bicarbonate-, sodium-, chloride- and sulphate-rich as well as magnesiac) for only 3 weeks, with the second lowest bicarbonate and the highest chloride content among the waters mentioned above containing these ions. Also, no modulation was observed upon fasting circulating sodium, potassium and magnesium although chloride increased [65]. Kiss et al. found no BP modulation in healthy women but a time-dependent increase in circulating and urinary magnesium excretion was identified after the consumption of a magnesium-rich mineral water for 4 weeks [49]. Nevertheless, if the populations of these studies had higher BP values, BP modulation (most probably a decrease) might have occurred as a consequence of mineral water intake [49,61,63,64,65,66].

Overall, only 4 [56,57,62,63] out of the 12 studies argued above analyzed the hormonal and enzymatic BP regulation [101,103,104,105]. Luft et al. reported a lack of effect of both waters tested (sodium chloride-rich control water and bicarbonate- and sodium-rich as well as magnesiac mineral-rich water) upon fasting circulating aldosterone and catecholamines (dopamine, epinephrine and norepinephrine); nevertheless, they induced a significant decrease of fasting circulating renin activity only in the hypertensive subset of participants, after 7 days [56]. Schorr et al. observed no effect upon circulating renin activity as well as atrial natriuretic factor, aldosterone and catecholamines for any of the mineral waters tested (low-mineral/low-sodium mineral water, bicarbonate-, sodium-, chloride- and sulphate-rich mineral-rich water and bicarbonate- and sodium-rich as well as magnesiac mineral-rich water), at the end of 4 weeks [62]. As already mentioned, these 2 studies occurred within a low-sodium diet environment. Interestingly, and in contrast to Schorr’s results [62], a Cochrane review revealed that a low-sodium daily intake increases circulating renin activity as well as aldosterone, epinephrine and norepinephrine [83]. Surprisingly, considering the absence of modulation of catecholamine levels found by Schorr et al. [62], a relevant increase of hepatic catechol-O-methyltransferase activity was reported in Wistar Han rats after the intake of NaHCO_3_^−^-rich mineral-rich water for 7 weeks [106]. Pereira et al. described that the ingestion of a NaHCO_3_^−^-rich mineral-rich water for 8 weeks seemed to protect against the increasing pattern of fasting circulating aldosterone induced by fructose consumption (in this protocol, a short fasting period was performed) [57]. Toxqui et al. observed a tendency to a decrease of fasting circulating aldosterone after the intake of a NaHCO_3_^−^-rich (and chloride-rich) mineral-rich water for 8 weeks [63]. These 4 chronic studies revealed no consistent modulation and relation between circulating aldosterone and urinary sodium excretion [56,57,62,63]. However, significant acute effects upon circulating aldosterone were described with 3 similar NaHCO_3_^−^-rich (also chloride-rich) mineral-rich waters [2 being the same used by Schoppen et al. [61] and Pérez-Granados et al. [58] and 1 similar to the ones from Toxqui et al. [63], Schoppen et al. [61] and Pérez-Granados et al. [58] studies] and an association with urinary sodium excretion was proposed [107,108]. In the two cross-over trials, in healthy postmenopausal women [107] and healthy younger women [108], the authors found a significant decrease in circulating aldosterone 120 min after mineral-rich water ingestion, with and without a meal [107,108]; water intake significantly increased urinary sodium excretion in 7-h urine [107]. The two mineral-rich waters, also fluorurate, tested in this last study, by Schoppen et al., behaved similarly but with the lower sodium content mineral-rich water showing a non-significant impact versus low-mineral content control water; no changes in urinary potassium, chloride and bone mineral excretion and urinary pH were observed [107].

The renin–angiotensin–aldosterone system plays a central role in the regulation of body water and salt balance as well as arterial BP, with its activity being controlled by renin. Considering sodium, and its role in arterial BP regulation, aldosterone prevents sodium renal loss while atrial natriuretic factor inhibits renin and aldosterone secretion and decreases sodium renal reabsorption (as it increases glomerular filtration rate) [104]. Catecholamines decrease kidney function. Bicarbonate (through metabolic acidosis/diet-induced acid load counteraction) and fluoride ions suppress aldosterone secretion [103,109] and inhibit sodium chloride reabsorption [105], respectively. The effects observed in the short-term upon (fasting and postprandial) circulating aldosterone and urinary sodium reabsorption might help BP regulation in the long-term [63]. Magnesium modulates the synthesis and secretion of aldosterone [9].

As these 12 studies vary in terms of the design and duration, diet and population size and characteristics, in addition to the type and amount of water consumed and BP-related mechanisms evaluated (Table 1), it is challenging to establish and characterize the profile of BP modulation by mineral(-rich) water ingestion.

### 4.2. Metabolic Syndrome Features—Lipid Profile

Increased fasting circulating total-triglycerides [≥ 150 mg/dL (or receiving drug therapy for hypertriglyceridemia)] and reduced fasting circulating HDL-cholesterol [< 40 mg/dL in men or < 50 mg/dL in women (or receiving drug therapy for reduced HDL-cholesterol)] are two other important features for MetSyn clinical diagnosis [1]. Adults with both these impaired levels have increased risk of incident coronary heart disease and stroke [110] which, as for hypertension, makes them an imperative preventable cause of death. Over 20% of patients from the population included in the European study on cardiovascular risk prevention and management in daily practice (EURIKA study) have either high fasting circulating total-triglycerides (≥ 200 mg/dL) or low fasting circulating HDL-cholesterol (< 40 mg/dL in men or < 50 mg/dL in women) levels [111,112]. In Latin America, (a) the prevalence of low fasting circulating HDL-cholesterol levels ranges from 34.1% in the CESCAS (*Centro de Excelencia en Salud Cardiovascular para el Cono Sur*) I study (< 40 mg/dL) to 53.3% in the Latin American consortium of studies in obesity (LASO study) (40 mg/dL in men and 50 mg/dL in women), presenting different frequencies between men and women, and (b) the prevalence of elevated fasting circulating total-triglycerides varies from 26.5% in the LASO study (≥ 150 mg/dL) to 31.2% in the National Health Survey of Chile (≥ 150 mg/dL), being more prevalent in men than in women [113,114,115].

#### Metabolic Syndrome Features Modulation by Mineral Water Consumption—Lipid Profile

A Spanish team of researchers led by Vaquero studied the effect of the same or quite similar mineral-rich waters on different populations using different design approaches upon lipid profile as well as lipid profile-related parameters [58,61,63,68,69,116]. After 8 weeks of treatment, a quite similar beneficial impact of NaHCO_3_^−^- and chloride-rich mineral-rich waters upon fasting circulating lipid profile and indexes of cardiovascular risk was observed in healthy postmenopausal women, with low-sodium diet (here the mineral-rich water was also fluorurate) [61], and moderately hypercholesterolemic young adults [58]. A significant decrease of total- and LDL-cholesterol as well as total-cholesterol/HDL-cholesterol and LDL-cholesterol/HDL-cholesterol ratios was found but no effect upon total-triglycerides and ApoAI was observed in both populations [58,61]; however, a significant decrease of ApoB was shown only in moderately hypercholesterolemic young adults [58]. Although a beneficial modulation of HDL-cholesterol was disclosed in both studies [58,61], it was significant only for the healthy postmenopausal women [61]. In these two interventions, control low-mineral and mineral-rich waters were tested consecutively [58,61]. Nevertheless, when a similar NaHCO_3_^−^- and chloride-rich mineral-rich water was consumed by moderately hypercholesterolemic young adults, also for 8 weeks but in a cross-over design, no differences were disclosed versus the control low-mineral water; however, a beneficial influence upon the fasting circulating lipid profile was observed with both waters [63]. It was hypothesized that the reduction of saturated fat intake within the free-living diet consumed when drinking the control low-mineral water (the opposite trend was observed when drinking the mineral-rich water) counterbalanced the possible specific favourable mineral-rich water effects [63]. Additionally and oppositely, Schoppen et al. observed, in healthy postmenopausal women, a significant beneficial mineral-rich water effect upon postprandial total area under the curve of circulating total- and chylomicron-triglycerides as well as postprandial peak concentration of circulating total-triglycerides [circulating chylomicron-cholesterol showed a similar but less intense and non-significant overall pattern of variation (both for postprandial total area under the curve and postprandial peak concentration)] [68]. Two similar NaHCO_3_^−^- and chloride-rich as well as fluorurate mineral-rich waters were tested by Schoppen et al. [68] in a cross-over design, one being the NaHCO_3_^−^-and chloride-rich as well as fluorurate mineral-rich water mentioned above [61]. In line, a protective effect upon postprandial lipaemia was found in moderately hypercholesterolemic young adults by Toxqui et al. with a NaHCO_3_^−^- and chloride-rich mineral-rich water (the same tested in [58]), in a cross-over design: a significantly lower increase of circulating total-triglycerides was observed that associated with a significantly lower increase of circulating cholecystokinin and significantly higher gallbladder volume, peak contraction amplitude and area under the curve as well as significantly lower gallbladder ejection fraction [69]. Consumption of the mineral-rich water without the standard fat-rich meal (versus control-water) did not significantly affect any of the studied parameters [69]. The water effect in these two studies seemed to be stronger in the healthy postmenopausal women than in the younger moderately hypercholesterolemic subjects as postprandial circulating total-triglycerides were reduced in 72% of the healthy postmenopausal women versus 66% of the moderately hypercholesterolemic young adults (the same fat-rich meal was consumed with the mineral-rich waters) [68,69,116].

These results concerning cholecystokinin and gallbladder-related parameters are consistent with a lower bile salts release during the postprandial period which, in turn, contributes to a reduction in lipid hydrolysis/digestion and absorption [69,117,118,119] and explains, almost completely, the highly complementary fasting and postprandial results obtained for the lipid profile and indexes of cardiovascular risk [58,61,68,69]. Cholecystokinin release depends, among other factors, on the gastric pH and gastric and pancreatic lipases activity. These parameters can be negatively modulated, as a consequence of the alkalinisation induced by the ingestion of mineral(-rich) waters (for example those mentioned in the paragraph above), and lead to the weakening of cholecystokinin release [58,61,63,68,69,120,121,122,123,124,125]. In line, this alkalinisation can also negatively interfere with fatty acid and cholesterol absorption from the already disturbed micellar solubilization [58,68,72]. Hence, altogether, these mechanisms explain the lowering of postprandial circulating total- and chylomicron-triglycerides and chylomicron-cholesterol and fasting circulating total- and LDL-cholesterol. Unexpectedly, a modulation of fasting circulating triglycerides was not observed despite a correlation between their levels and the magnitude of postprandial lipemia has been reported [58,61,63,66,126,127]. The results of the Spanish team mirror the fact that lower postprandial circulating triglycerides favor higher fasting circulating HDL-cholesterol [127,128] and, curiously and accordingly, the increase of fasting circulating HDL-cholesterol was statistically significant only in the more postprandial circulating total-triglyceride-lowering responsive population sample, the healthy postmenopausal women [58,61,68,69,116].

Curiously, Corradini et al. noticed the reduction of fasting gallbladder volume and the increase of fasting circulating total bile acids, without any change in the lipid profile, in gallstone-free postmenopausal women (with functional dyspepsia and/or constipation) drinking a sulphate- and bicarbonate-rich as well as magnesiac, calcic and fluorurate mineral-rich water [73]. The shorter intervention time when compared to Schoppen et al. (12 days versus 8 weeks) might have not allowed the demonstration of a beneficial modulation of the lipid profile [61,73]. By contrast, as a consequence of significantly increased gallbladder contraction and total fecal bile acids excretion, Capurso et al. reported a significant decrease of fasting circulating total-cholesterol, LDL-cholesterol and ApoB as well as total-cholesterol/HDL-cholesterol ratio, but without modification of HDL-cholesterol, after the ingestion of mineral-rich water (bicarbonate-, sodium-, chloride- and sulphate-rich as well as magnesiac) for 3 weeks by moderately hypercholesterolemic subjects. Similar to Vaquero´s team studies [58,61], and also in a consecutive design, fasting circulating total-triglycerides and apoAI did not change [65]. Cantalamessa et al. found a similar impact upon the fasting circulating lipid profile (reduction of total- and LDL-cholesterol as well as of total-cholesterol/HDL-cholesterol, total-cholesterol/LDL-cholesterol and HDL-cholesterol/LDL-cholesterol ratios but without any change in HDL-cholesterol and total-triglycerides) and total fecal bile acid elimination in hypercholesterolemic pathogen-free male CD rats, in a dietary parallel intervention that lasted 4 months with mineral-rich water (bicarbonate-, sodium-, chloride- and sulphate-rich as well as sulphurous, calcic, magnesiac and fluorurate) [70]. A decrease in the enterohepatic circulation of bile acids owing to an increase in their fecal loss (due to increased bile production, gallbladder contraction/emptying and/or intestinal propulsion as well as intestinal formation of insoluble and unabsorbable soaps from bile acids) stimulates the liver to produce more bile acids from cholesterol leading to a decrease of circulating total- and LDL-cholesterol and an increase of circulating HDL-cholesterol (the latter, through reverse cholesterol transport, provides cholesterol to the liver to be converted to bile acids) [58,61,65,68,70]. In agreement, several other authors making use of mineral(-rich) waters with quite distinct compositions also found the bile production, gallbladder contraction/emptying and/or intestinal propulsion/transit increased response, most probably due to both specific ion profile and osmolar concentration of mineral(-rich) waters [51,58,68,69,72,129,130,131,132,133,134,135,136,137,138]. The health status does not seem to interfere in this response [51,58,65,68,69,70,72,129,130,131,132,133,134,135,136,137,138]. 

Unlike Toxqui et al. using a 62% fat-rich meal [69], Zair et al. did not find a response of the postprandial lipid profile when combining a 42% fat-rich meal with a strongly bicarbonated and sodium- and chloride-rich mineral-rich water in moderately hypercholesterolemic men (also in a cross-over design): the lower fat % precluded the appearance of a high postprandial lipemia to be modulated by the ingestion of the mineral-rich water, which highlights, once more, the role of diet composition in this type of intervention studies [66]. Similarly to Toxqui et al. [63], in corresponding metabolically dysfunctional individuals on 8-week treatment period in a cross-over design, there were no differences between the control low-mineral and mineral-rich waters, although a significant decrease in fasting circulating total- and VLDL-triglycerides (and a tendency to decrease fasting circulating VLDL-cholesterol) was observed with the latter [66]. The distinct water composition justifies why on this study, unlike on other studies mentioned so far, triglyceride- instead of cholesterol-related (not considering HDL) parameters were significantly modulated [66]. Accordingly, and likewise in line with a very low Cl^−^/HCO_3_^−^ ratio in the mineral-rich water tested, Pereira et al. described lower fasting circulating total-triglycerides increase and no change of cholesterol-related parameters in a MetSyn animal model, with a NaHCO_3_^−^-rich mineral-rich water in a dietary parallel intervention that lasted 8 weeks [57]. Oppositely, higher Cl^−^/HCO_3_^−^ ratios were present in the waters chronically tested by Vaquero´s team [58,61,63], Capurso et al. [65] and Cantalamessa et al. [70]. Furthermore, in hiperlipidemic adults, Aslanabadi et al., in a dietary parallel intervention that lasted 1 month, found no differences between the control low-mineral and mineral-rich waters (the latter being bicarbonate-rich and calcic; with no information regarding its chloride content), both showing a beneficial impact [72].

However, no effects at all were observed upon the circulating lipid profile by Murakami et al. and Schorr et al. with mineral-rich waters, respectively, in healthy subjects drinking bicarbonate-, sodium- and sulphate-rich as well as magnesiac and calcic mineral-rich water for 2 weeks (consecutive alternate design) [71] and in elderly healthy normotensives under a low-sodium diet and drinking NaCl-rich mineral water (also bicarbonate- and sulphate-rich) or NaHCO_3_^−^-rich mineral water (also magnesiac) for 4 weeks (cross-over design) [62]. As Capurso et al. found a modulation of the lipid profile after 3 weeks of treatment [65], the length of the intervention should not have been the cause of the lack of effect just exposed. Nevertheless, with Pérez-Granados et al. the duration of the treatment seemed to have played a role in the magnitude of the effects observed as the reduction pattern visible at the 4th week for fasting circulating total- and LDL-cholesterol as well as total-cholesterol/HDL-cholesterol ratio became significant at the 8th week [58]. Interestingly, Schorr et al. also did not find any change of the circulating lipid profile in the low-mineral/low-sodium control water + low-sodium diet [62], although an increase of circulating cholesterol and triglycerides has been associated with a low sodium intake [83]. This association might have contributed to the higher magnitude of the mineral-rich water effects upon the fasting circulating lipid profile observed in Schoppen et al. [61] versus Pérez-Granados et al. [58]. The same for the higher fluoride content [139] present in the mineral-rich water tested by Schoppen et al. [61] versus Pérez-Granados et al. [58]. On the other hand, quite similar effects upon the postprandial lipemia were observed by Schoppen et al. when testing two NaHCO_3_^−^-rich, chloride-rich and fluorurate mineral-rich waters that differed 5.6 times in their fluoride content [68]. Nevertheless, it should be mentioned that rats’ exposure, for 7 weeks, to fluoride through drinking water in concentrations much higher than the ones found in the mineral-rich waters included in this review was associated with disturbance of lipid homeostasis as well as induction of pro-inflammatory and oxidative processes [140].

Among the 5 studies presenting improvement of the lipid parameters included in MetSyn definition [57,58,61,68,69], bicarbonate, sodium, chloride and fluoride were present in relevant amounts [47]. Interestingly, within the 13 studies, age, diet composition as well as health and nutritional status of the individuals [the latter underlying the chronic or acute characteristics of the study: fasting (after a given treatment period) or postprandial states, respectively], along with the duration of the treatment, seem to have conditioned the results (Table 2). As for BP, it is not easy to determine the pattern of lipid profile modulation by mineral(-rich) water ingestion.

### 4.3. Metabolic Syndrome Features—Glucose

Fasting circulating glucose ≥ 100 mg/dL (or receiving drug therapy for hyperglycemia) constitutes a condition considered for MetSyn clinical diagnosis [1]. People presenting an impaired glucose regulation are at an increased risk of developing T2DM. In developed Europe, over one in five people meet the criteria for either impaired glucose tolerance, impaired fasting glucose or both [141]. Diabetes is a major cause of blindness, kidney failure, myocardial infarction, stroke and lower limb amputation, as well as other long-term consequences that impact significantly on life quality. The WHO estimated that diabetes was the seventh leading cause of death in 2016 [81,142,143,144]. The International Diabetes Federation projected that, globally in 2015, 415 million adults aged 20–79 years (1 in 11 individuals) had diabetes mellitus, over 90% presenting T2DM, and that the number will to rise to 642 million by 2040 [145]. Again, as for hypertension and impaired lipid profile, altered glucose regulation should be actively prevented and treated. 

#### Metabolic Syndrome Features Modulation by Mineral Water Consumption—Glucose

The Spanish team of researchers led by Vaquero also studied the effect of the same or quite similar mineral-rich waters on different populations using different design approaches upon glucose as well as glucose homeostasis-related parameters [58,61,63,69,75]. As in the previous section, population characteristics, diet composition and nutritional status of the individuals conditioned the drawing of generalized conclusions. Toxqui et al. observed a tendency to overall lower postprandial circulating insulin and a lack of effect upon postprandial circulating glucose in moderately hypercholesterolemic young adults that ingested a fat-rich meal along with a NaHCO_3_^−^- and chloride-rich mineral-rich water (the same tested in [58]) [69]. Consumption of the mineral-rich water without the standard fat-rich meal (versus control-water) did not significantly affect any of the studied parameters [69]. Nevertheless, for the same fat-rich meal but in healthy postmenopausal women, Schoppen et al. disclosed, for 2 similar NaHCO_3_^−^- and chloride-rich as well as fluorurate mineral-rich waters (the water richer in fluoride being the same used in [61]) lower postprandial circulating insulin only at the end of the evaluation period (at its initial segment, the opposite occurred) versus control water. For both time points, higher intensity effects were observed with the water richer in fluoride (attaining significance at the last assessment). Postprandial insulinemia depended also on volunteer´s HOMA index distribution values [75]. Interestingly, lower fluoride water content as well as body mass index and baseline insulin values occurred in the former study [69]. No relevant impact upon glycemia was observed in these 2 aforementioned short-term interventions (it should be highlighted that the fat-rich meal consumed had a low % of both complex carbohydrates and added sugar) [69,75]. In 2 out of 3 chronic design studies from the Vaquero team with moderately hypercholesterolemic young subjects drinking similar mineral-rich waters, already mentioned in the BP and lipid profile sections of this review, a lack of effect upon fasting circulating insulin and a decrease of fasting circulating glucose were observed [58,63]. Although significance was reached for fasting circulating glucose in the Toxqui et al. trial, it was ascribed to the reduction of soft drink and fruit juice consumption throughout the intervention as a significant decrease of that variable was also noticed with the control low-mineral water [63]. In the third chronic design study, in healthy postmenopausal women, the Vaquero team disclosed a significant decrease of fasting circulating glucose with the NaHCO_3_^−^- and chloride-rich as well as fluorurate mineral-rich water [61]. As for the lipid profile, the effects upon fasting circulating glucose were more intense in Schoppen et al. [61] versus Pérez-Granados et al. [58]. This might have resulted from the quite distinct fluoride content of the mineral-rich waters (being the main difference between these 2 waters: lower in Pérez-Granados et al. [58]) and the low-sodium diet of the Schoppen et al. volunteers [61]. Nevertheless, it should pointed out that fluoride modulation of insulin secretion, action and clearance is still not completely clarified [75,146]. In contrast, NaCl restriction has been revealed to deteriorate glucose tolerance and high NaCl intake to regulate glucose homeostasis (the latter through adiponectin production) [147,148,149,150]. 

Nevertheless, and once more as before, Schorr et al. did not find any change of circulating glucose and insulin, not even after an oral glucose load, among treatments and versus baseline, despite the low or high sodium (with two distinct anions) intake, in healthy normotensive subjects (included in the counting of postprandial studies) [62]. The absence of influence upon both fasting and postprandial circulating glucose was also observed by Zair et al. in moderate hypercholesterolemia when testing a strongly bicarbonated as well as chloride- and sodium-rich mineral-rich water [66]. Here, the percentage of energy from carbohydrates in the postprandial study meal was higher than in Toxqui et al. [69] and Schoppen et al. [75] (45 versus 30%). In those 2 trials, intervention time does not seem to have played a role in absence of modulation [62,66] as Murakami et al. found a beneficial inflection of fasting circulating glycoalbumin and glucose (with significance only for the former) in healthy subjects that ingested a bicarbonate-, sodium- and sulphate-rich as well as magnesiac and calcic mineral-rich water for a shorter period. Extraordinarily, a valuable reduction of fasting circulating amino acids usually increased in hyperinsulinemia, but without any changes in HOMAR or circulating insulin, was observed [71]. Nevertheless, the ingestion of NaHCO_3_^−^-rich mineral-rich water counteracted the negative effects of fructose ingestion, by Sprague–Dawley rats, not only upon circulating glucose but also upon circulating insulin and leptin (short fasting period was done) as well as in insulin sensitivity index [57]. The beneficial modulation of the redox status, insulin and glucocorticoid signaling, Sirt1 protein expression and endoplasmic reticulum stress in the liver and/or adipose tissue was achieved in this animal protocol explaining the positive impact upon fasting circulating lipid profile and glucose, besides BP. Glucocorticoids contribute to dyslipidemia, hyperglycemia and insulin resistance. Sirt1 has a role in glucocorticoid signaling. The disruption of endoplasmic reticulum homeostasis and impaired redox status can cause insulin resistance, which associates with hypertension [13,57,97,151,152,153]. Furthermore, El-Seweidy et al. disclosed that the ingestion of a chloride-, sodium- and sulphate-rich as well as sulphurous, calcic and fluorurate mineral-rich water, for 7 weeks, improved glucose homeostasis in rats with streptozotocin-induced diabetes [74]. Interestingly, in this animal protocol, the sulphurous mineral-rich water counteracted the enhanced expression of cardiac NF-κB, profibrogenic and apoptotic parameters, most probably by restoring the redox balance, concomitantly with an improved histology (quite similar results were obtained with NaHS, an exogenous H_2_S donor) [74]. 

Fluoride, chloride, sodium, bicarbonate, sulphate/H_2_S/H_2_S donors, magnesium and calcium are present in relevant amounts [47,74] in the mineral-rich waters linked to positive effects upon glucose [57,58,61,71,74]. Overall, the protective effects of H_2_S upon MetSyn and its associated complications have been related to its beneficial modulation of insulin and insulin growth factor formation, inflammation, redox status, apoptosis, platelet aggregation, thrombolysis, vasodilation and vasorelaxation processes and vascular endothelial growth factor expression and secretion in addition to the nitric oxide and H_2_S cross-talk [53,74,154,155]. In this regard, an increased release of the anti-inflammatory cytokine interleukin 10 has been observed in primary human monocytes incubated with sulphurous thermal water and in airway disease patients’ saliva undergoing thermal treatments with the same water. Curiously, this latter increase correlated positively with salivary catalase activity [156]. In line, the ingestion of 0.5 L/day of a sulphurous mineral-rich water (also bicarbonate- and sulphate-rich, calcic, magnesiac, fluorurate and with carbon dioxide) by healthy subjects, for 2 weeks, significantly decreased both circulating lipid and protein oxidation products (malondialdehyde, protein carbonyls and advanced oxidation protein products) and significantly increased circulating total antioxidant capacity as well as total thiols [157]. The ingestion of 2 distinct sulphurous mineral-rich waters (also bicarbonate-, sodium-, chloride- and sulphate-rich, calcic, magnesiac, fluorurate and with carbon dioxide), for 2 weeks, decreased circulating reactive oxygen species in healthy rats [158]. Interestingly, due to its magnesium content, sulphurous mineral water may combat the state of hypomagnesaemia often found in diabetes [9,74]. Magnesium is needed for β-cell function, is crucial for insulin signalling (is essential for the insulin-insulin receptor interaction, the affinity of the insulin receptor tyrosine kinase for ATP and the autophosphorylation of the β-subunits of the insulin receptor), has anti-inflammatory and anti-oxidant properties, plays a role in the regulation of glucocorticoid production and is a co-factor in intra- and extracellular lipid metabolism [9,28,91,99,159,160,161,162,163]. Bicarbonate, through correction of metabolic acidosis/counteraction of diet-induced acid load, improves insulin sensitivity and decreases glucocorticoid production [9,20,22,25,75,162]. Dietary calcium has been linked to anti-inflammatory and anti-oxidant properties, lipid metabolism towards its utilization, insulin sensitivity, increased thermogenesis, reduced glucocorticoid production and increased Sirt1 protein expression [9,164]. In accordance, calcium deficiency has been linked to insulin resistance [71].

Elevated fasting circulating insulin concentrations (or insulin resistance) are independently associated with an exacerbated risk of hypertension in the general population [153]. From the 5 studies in which BP was positively modulated [56,57,58,59,60], only 2 measured fasting circulating insulin [57,58] with a simultaneous beneficial modulation of fasting circulating insulin and BP reported in [57]. Only 2 studies revealed a positive modulation of BP as well as fasting circulating lipid profile and glucose homeostasis [57,58]. Among the 8 studies that simultaneously appraised the effects of mineral-rich water consumption upon the lipid profile and glucose (in addition to several related parameters) [57,58,61,62,63,66,69,71], only 3 revealed concurrent positive impact upon both MetSyn features [57,58,61] and only 2 exposed concomitant beneficial modulation of circulating total-triglycerides and insulin/insulin sensitivity index [57,69], which are strongly correlated [15] (Table 1, Table 2 and Table 3).

### 4.4. Metabolic Syndrome Features—Waist Circumference

Elevated WC, with population- and country-specific cut-off points, is also a feature considered for MetSyn clinical diagnosis [1]. According to the WHO, the prevalence of obesity nearly tripled between 1975 and 2016. In 2016, (a) more than 1.9 billion adults (18 years and older) were overweight and 650 million were obese, and (b) 39% of adults were overweight and 13% were obese [165,166]. Again, as for hypertension and impaired lipid profile and glucose homeostasis, overweight/obesity and elevated WC and body mass index should be actively prevented and treated.

#### Metabolic Syndrome Features Modulation by Mineral Water Consumption—Waist Circumference

Cantalamessa et al. detected that pathogen-free male CD rats consuming a hypercholesterolemic diet and drinking a mineral-rich water (bicarbonate-, sodium-, chloride- and sulphate-rich as well as sulphurous, calcic, magnesiac and fluorurate) had a higher body weight increase than rats with access to the same diet but ingesting tap water. However, it should be mentioned that these 2 groups of rats grew significantly less than the controls [70]. A similar protective effect against body weight loss was observed in rats with streptozotocin-induced diabetes when drinking a chloride-, sodium- and sulphate-rich as well as sulphurous, calcic and fluorurate mineral-rich water [74]. By contrast, Corradini et al. spotted that in postmenopausal women the consumption of a sulphate- and bicarbonate-rich as well as magnesiac, calcic and fluorurate mineral-rich water avert body weight gain, despite increased food consumption (around the double quantity of pasta, meat and vegetables but half of bread, with no other modification) [73]. This could be explained by (a) a putatively increased gastrointestinal emptying/intestinal transit (as a consequence of the disclosed increase of daily bowel movements number) as well as the noticed improvement of gallbladder motility as, together, these mechanisms likely increase the frequency of bile acid enterohepatic circulation and fecal losses, with a secondary stimulation of primary bile acids hepatic synthesis and (b) a putatively increased energy expenditure in brown adipose tissue and muscle (through promotion of intracellular thyroid hormone activation secondary to the activation of the TGR5-signaling pathway) induced by the observed increase of circulating total bile acids (which have been recognized as important modulators of whole-body metabolism) [73]. On the other hand, the Spanish team perceived no relevant/direct effects of mineral-rich water ingestion upon food intake or body weight, although the lower cholecystokinin levels observed might induce higher food intake (cholecystokinin inhibits gastric emptying and is a satiety signal) [58,61,63,69]. The other studies within this section of the review showed no specific impact upon body weight, body mass index and food intake (Table 4) [56,57,64].

Calcium, present in a relevant amount [47] in the mineral-rich water tested by Corradini et al. [73], has been associated with weight loss [167] and weight loss has been related to an improvement of the inflammatory state observed in obesity [168]. However, it should be mentioned that the combination of a magnesium deficit with an increase in calcium intake may allow the attenuation of the calcium channel-blocking effect of magnesium leading to an increased calcium entry into immunocompetent cells stimulating an inflammatory response [169]. Additionally, besides inflammation, a strong physiological/cellular link between a rising intracellular ratio of calcium to magnesium and aspects of metabolic syndrome, including hypertension, hyperinsulinemia, insulin resistance and left ventricular cardiac hypertrophy, has been described [170]. Considering that postmenopausal women were included in some of the studies discussed in this review (as for example in [73]) and menopause is a risk factor for osteoporosis [171], the consumption of mineral waters with calcium may impair bisphosphonates bioavailability [37,172].

## 5. Conclusions

Mineral water consumption represents not only a good source of specific minerals/elements, active ions and molecules but also an adequate tool against diet-induced acid-load. As such, from this review, we can highlight the need to control effectively and acknowledge acid-base balance and minerals/elements, active ions and molecules status in the body as well as the composition and acid-load capacity of the diet of the volunteers included in the studies that evaluate mineral water consumption impact upon MetSyn. This is most crucial because mineral water consumption could be happening in different baseline backgrounds and, so, revealing different results. From one extreme to the other, mineral water consumption could be either (a) compensating deficiencies in the body and diet and/or correcting acid-base imbalance in the body and reducing diet acid-load capacity, or (b) supplementing an already adequate body status and dietary ingestion, with further alkalinisation of the acid-base balance in the body and/or further reduction of acid-load capacity of the diet. In between, it could be mitigating or increasing disproportions in mineral/element/active ion/molecule ratios in the body and diet. In addition, this review also shows that the results of studies on mineral water consumption are dependent on the maintenance, or not, of a given diet along the corresponding study as well as on its macronutrient composition.

Globally, ingestion of mineral waters might be beneficial upon BP regulation when a dysfunction in metabolism and/or mineral/elements homeostasis exits. The same has become evident for lipid profile and glucose homeostasis, but more independently of the health status than revealed for BP.

Further studies are warranted for unravelling the full spectrum of individuals that could benefit from mineral water consumption in terms of MetSyn prevention and/or treatment and for fully determining the mechanisms involved in these actions, but in which the detailed dietary and health backgrounds should be evaluated and controlled.

## Figures and Tables

**Table 1:** The influence of mineral water consumption upon blood pressure.

Ref	Population and Study Type; Water Classification	Aim and Intervention	Results
Studies in animal models.			
[57]	21 Adult male CD Sprague–Dawley rats (388–483 g). Randomized intervention, including 3 groups in parallel.	To investigate the effects of mineral water^⊗^ consumption in 10% fructose-fed Sprague–Dawley rats (Table 2, Table 3 and Table 4). The animals were divided into 3 groups (n = 7/group), fed with standard laboratory chow diet and different drinking solutions, for 8 weeks. (a) Group CONT: rats drinking tap water; (b) group FRUCT: rats drinking 10% fructose in tap water; and (c) group FRUCTMIN: rats drinking 10% fructose in mineral water (Pedras Salgadas^®^). This mineral water was mainly rich in bicarbonate (2013 mg/L) and sodium (591 mg/L); also containing 30.8 mg/L chloride and 6.4 mg/L sulphate and presenting higher potassium (29.9 mg/L), magnesium (26.2 mg/L) and calcium (92.5 mg/L) content than tap water.	SBP, DBP and HR evolution over time (over the dietary intervention period) appeared to be protected from fructose effects by mineral water ingestion until approximately half of the dietary intervention period. SBP significantly increased with time in both fructose groups versus CONT group. DBP in FRUCTMIN rats tended to increase with time versus CONT rats. Between weeks 1 and 5, FRUCT rats had a significantly higher HR than CONT rats. HR significantly increased with time in FRUCTMIN versus CONT rats. Circulating aldosterone seemed to increase in FRUCT versus CONT (mineral water ingestion seemed to counteract this pattern). Between weeks 0 and 6, FRUCTMIN rats presented significantly higher urinary sodium versus CONT and FRUCT rats. FRUCTMIN and FRUCT rats showed significantly and similarly lower circulating magnesium than CONT rats. No differences were found in circulating chloride, phosphorous, sodium, calcium and potassium among the 3 groups. No differences in circulating uric acid; CONT rats presented significantly higher circulating urea than FRUCT and FRUCTMIN rats.
	^⊗^ Water classified as mineral-rich, bicarbonate-rich and sodium-rich.		
Studies in humans.			
[61]	18 Postmenopausal women (amenorrheic ≥ 1 y, aged 45–59 y), healthy and not obese (BMI < 30 Kg/m^2^). With exclusion criteria. Clinical trial, well-controlled sequential design, non-blinded.	To investigate the effects of mineral water^⊗^ consumption in healthy postmenopause (Table 2, Table 3 and Table 4). The intervention comprised 2 consecutive periods of 2 months each. Participants consumed, as supplement to their usual diet, 1 L/day of control low-mineral water (CLMW) during the first period and 1 L/day of carbonic sodium-bicarbonate mineral water (CSBMW) during the second period. CLMW: 71.1 mg/L bicarbonate, 9 mg/L sodium, 5.7 mg/L chloride, 25.2 mg/L calcium, 2.7 mg/L magnesium, 1.4 mg/L potassium, 0.2 mg/L fluoride and 15.7 mg/L sulphate; without carbonic gas. CSBMW was mainly rich in bicarbonate (2094.4 mg/L), sodium (1116.5 mg/L) and chloride (583.0 mg/L) and contained carbonic gas; its content in calcium, magnesium, potassium, fluoride and sulphate being 43.6, 5.7, 54.7, 7.9 and 49.9 mg/L, respectively. Both waters were from Vichy Catalán^®^. Diet was controlled, characterized and analysed; compliance with treatments was checked. Determinations were performed at the beginning and end of the CLMW and CSBMW periods. Blood samples were collected after 12 h of overnight fasting.	SBP and DBP did not change during the study.
	^⊗^ Water classified as mineral-rich, bicarbonate-rich, sodium-rich, chloride-rich and fluorurate.		
[58]	18 Young volunteers (8 men and 10 women), aged > 18 and < 40 y [29 ± 8 y (mean ± SD)], moderately hypercholesterolemic (total- cholesterol > 200 mg/dL and LDL-cholesterol > 100 mg/dL); BMI 24.38 ± 4.24 Kg/m^2^ (mean ± SD). With exclusion criteria. Clinical trial, controlled sequential design, non-blinded.	To investigate whether the effects of mineral water^⊗^ intake previously observed in healthy postmenopause were confirmed in moderately hypercholesterolemia (Table 2, Table 3 and Table 4). Intervention lasted 2 consecutive 8-week periods. Participants consumed, as supplement to their usual diet, 1 L/day of CLMW during the first period and 1 L/day of CSBMW during the second period. CLMW contained 104 mg/L bicarbonate, 8.7 mg/L sodium, 11 mg/L chloride, 33.4 mg/L calcium, 5.0 mg/L magnesium, 2.0 mg/L potassium, < 0.2 mg/L fluoride and 15.6 mg/L sulphate; without carbonic gas. CSBMW was mainly rich in bicarbonate (2120 mg/L), sodium (1102 mg/L) and chloride (597 mg/L); its content in calcium, magnesium, potassium, fluoride and sulphate being 32.0, 9.4, 49.5, 0.9 and 45.3 mg/L, respectively; 3.9 g/L carbonic gas. Both waters were from Vichy Catalán^®^. Diet was controlled, characterized and analysed; compliance with treatments was checked. Participants were instructed to maintain their regular habits as well as their normal diet and exercise level. Determinations were performed at baseline, at the end of the CLMW period and at weeks 4 and/or 8 of the CSBMW period. Blood samples were collected after a 12 h fasting period. Volunteers were instructed regarding the composition of their dinner on the evening before the analysis. At the end of both intervention periods, 24 h urine samples were collected.	Consumption of CSBMW for 4 weeks significantly decreased SBP, within normal limits, versus CLMW intake (but not for 8 weeks of ingestion); without significant differences in SBP between the fourth and the eighth weeks of CSBMW intake. DBP pressure remained constant during the entire study. Urinary pH and sodium and chloride were significantly higher with CSBMW versus CLMW ingestion, at the eighth week.
	^⊗^ Water classified as mineral-rich, bicarbonate-rich, sodium-rich and chloride-rich.		
[66]	12 Healthy men, moderately hypercholesterolemic (2.20–3 g/L), aged 20–60 y [40.6 ± 9.4 y (mean ± SD)], BMI 18.5–25 Kg/m^2^ [23.1 ± 2.2 Kg/m^2^ (mean ± SD)]. With exclusion criteria. Controlled, randomized, double-blinded, cross-over design, including 2 groups.	To analyze the effects of mineral water^⊗^ consumption, with or without a standardised meal, in moderate hypercholesterolemia (Table 2 and Table 3). The study consisted in 2 experimental periods with 8 weeks each separated by a 1-week wash-out period. Subjects consumed 1.25 L/day of 1 of the 2 sparkling mineral waters. Saint-Yorre^®^ (SY) water, with high-mineral content (20 fold more than the referent water); mainly rich in bicarbonate (4168 mg/L), sodium (1626 mg/L) and chloride (329 mg/L) and its content in potassium, calcium, magnesium and sulphate being 117, 85, 11 and 186 mg/L, respectively. Ogeu^®^ (referent water), with low-mineral content (183, 31, 48, 1, 48, 12 and 18 mg/L, respectively). Diet was controlled and characterized. Volunteers were asked not to deviate from their regular habits during the study. BP measurements and blood sampling were done in a first visit just before starting SY water or referent water consumption and in a second visit on the last day of SY water or referent water intake. Their effects were studied at fasting (T0) and/or during postprandial state (T30 min, T1 h, T2 h, T3 h, T4 h, T6 h and T8 h) after ingestion of a standardised meal and 0.5 L of water, at both visits for both waters.	No significant differences were found on SBP and DBP considering both treatments, both at basal or postprandial ^$^ states.
	^⊗^ Water classified as mineral-rich, bicarbonate-rich, sodium-rich and chloride-rich.		
[63]	64 Normotensive participants, 56% women, aged 18–45 y [30.7 ± 7.5 y (mean ± SD)], moderately hypercholesterolemic (total-cholesterol: > 200 and < 300 mg/dL), BMI 23.2 ± 2.8 kg/m^2^. With exclusion criteria. Randomized, single-blinded, cross-over, controlled trial, including 2 groups.	To assess the effects of mineral water^⊗^ intake in moderate hypercholesterolemia (Table 2, Table 3 and Table 4). The intervention consisted in two 8-week periods [one water tested/period, 1 L/day (consumed as part of the usual diet, with the 2 main meals)], separated by an 8-week washout period. Participants were allocated in 2 groups: (a) CLMW, that had 74.9 mg/L bicarbonate, 7.6 mg/L sodium, 4.8 mg/L chloride, 22.6 mg/L calcium, 2.8 mg/L magnesium, 1.7 mg/L potassium, 0.2 mg/L fluoride and 10.6 mg/L sulphate; without carbon dioxide; and (b) CSBMW was mainly rich in bicarbonate (2050 mg/L), sodium (1090 mg/L) and chloride (622 mg/L); its content in calcium, magnesium, potassium, fluoride and sulphate being 20.8, 5.8, 47.2, 0.73 and 50 mg/L, respectively; with carbon dioxide (4.5 g/L). Both waters were from Vichy Catalán^®^. Diet was controlled, characterized and analysed; compliance with treatments was checked. Participants were asked to maintain their regular habits as well as their normal diet, body weight and exercise level. Determinations were made at baseline and at the fourth and eighth weeks. Blood samples were collected after 12 h of overnight fasting.	SBP and DBP did not vary significantly throughout the trial. Circulating aldosterone did not vary significantly throughout the trial, although it tended to decrease with CSBMW, but not with CLMW intake. CSBMW consumption significantly increased urinary pH and decreased urinary calcium; CLMW intake did not change these parameters. Urinary potassium significantly decreased with time for both mineral waters while urinary sodium and phosphate remained stable.
	^⊗^ Water classified as mineral-rich, bicarbonate-rich, sodium-rich and chloride-rich.		
[56]	20 Participants, 10 men and 10 women: 10 mildly hypertensive (BP > 140/90 mmHg; 5 black + 5 white) and 10 normotensive (BP < 140/90 mmHg; 5 black + 5 white) subjects. Aged 36 ± 9 y (mean ± SD), 20–56 y. With exclusion criteria. Randomized, placebo-controlled, double-blinded, cross-over trial, including 2 groups.	To test the effects of sodium bicarbonate and sodium chloride ingestion (as drinking solutions) in normotensive and mildly hypertensive subjects consuming a low-sodium diet (Table 4). Each intervention phase lasted 11 days. In the first 4 days, participants ingested a fixed daily basal low-sodium diet aiming to achieve ion balance (60 mmol sodium, 60 mmol chloride, 60 mmol potassium and 14 mmol calcium; 80 g protein; fat and carbohydrates were adjusted for each participant). Then, keeping the same diet, they were assigned to drink 3 L/day, for 7 days, of a mineral water^⊗^ (Staatlich Fachingen^®^, 26.2 mmol/L sodium, 0.71 mmol/L potassium, 2.18 mmol/L magnesium, 3.04 mmol/L calcium, 4.23 mmol/L chloride and 33.03 mmol/L bicarbonate) or a control placebo aqueous solution (containing the same concentrations of all of the cations as the chloride salts, 36.07 mmol/L). In both regimens, total daily sodium consumption increased to 138 mmol. One month later (at least), the opposite regimen was followed. Food and liquids provided by the research team. Determinations were done on the fifth and twelfth day, after overnight fasting.	HR was not modulated by both treatments. Sodium chloride-containing control water did not influence BP. In mildly hypertensives, sodium bicarbonate-containing mineral water significantly decreased SBP but not DBP; no effect was observed in normotensives. Both drinking regimens significantly decreased circulating renin activity in mildly hypertensives but not in normotensives; no effects were observed upon circulating aldosterone, dopamine, epinephrine and norepinephrine. No differences were found in urinary sodium or potassium between drinking regimens (urinary sodium similarly increased with both waters; data not provided for potassium), BP groups and race. Urinary chloride consistently and significantly increased with sodium chloride-containing control water but not with sodium bicarbonate-containing mineral water; a similar significant pattern was observed for urinary calcium, although more weak (an effect of race and BP was also evident for calcium: greater in whites and mildly hypertensives than in blacks and normotensives, respectively). Urinary urate was similar in the 2 BP groups for both dietary oral interventions; however, for both drinking solutions, it was significantly greater in whites than in blacks. Sodium chloride-containing control water, versus sodium bicarbonate-containing mineral water, had significant, but modest, positive and negative effects on circulating chloride (in both BP groups) and bicarbonate (only in normotensives; non-significant similar pattern in hypertensives), respectively. Circulating sodium, potassium, calcium, phosphate, urate and albumin were not influenced by any of the waters tested.
	^⊗^ Water classified as mineral-rich, bicarbonate-rich, sodium-rich and magnesiac.		
[64]	17 Normotensive healthy participants (BP < 140/90 mmHg; 9 women and 8 men), aged 24–53 y (median 29 y); BMI 17.3–33.5 Kg/m^2^. With exclusion criteria. Randomized, non-blinded, cross-over study, including 2 groups.	To evaluate the effects of mineral water^⊗^ consumption in healthy normotensive subjects (Table 4). Each arm of the study lasted 7 weeks, with a 6-week washout period. Participants ingested 0.5 L/day of Água das Pedras/Pedras Salgadas^®^ or Vitalis^®^ (0.25 L in the morning and 0.25 L in the afternoon). Água das Pedras^®^, carbonic hypersaline (sodium and bicarbonate-rich) mineral water: 62.0 mg/L silica, 31.0 mg/L chloride, 2125.0 mg/L bicarbonate, 622.0 mg/L sodium, 28.0 mg/L magnesium, 103.0 mg/L calcium and 0.3 mg/L nitrate. Vitalis^®^, hyposaline water: 10.0 mg/L silica, 7.0 mg/L chloride, < 1.0 mg/L bicarbonate, 3.8 mg/L sodium, 0.7 mg/L magnesium, 0.4 mg/L calcium and nitrate 1.2 mg/L; without carbon dioxide. Diet was controlled, characterized and analysed; compliance with treatments was checked. Participants had no restrictions on food or other drinks ingestion but were asked to maintain their normal eating and drinking habits. Measurements were done at the beginning and end of each treatment.	No significant differences were observed between the effects of the 2 treatments upon SBP, DBP and HR. No significant difference from baseline values was found. None of the 2 treatments had any effect on urinary pH or sodium and potassium.
	^⊗^ Water classified as mineral-rich, bicarbonate-rich and sodium-rich.		
[62]	21 Healthy normotensive participants [men (n = 10) and women (n = 11)], aged 64.1 ± 3.6 y (mean ± SD; 60–72 y); BMI 26.1 ± 3.6 Kg/m^2^. With exclusion criteria. Randomized, placebo-controlled, double-blinded cross-over trial, including 3 groups.	To examine the effects of mineral water^⊗^ ingestion in healthy normotensive participants consuming a low-sodium diet (an oral glucose tolerance test was also done; Table 2 and Table 3). Each phase of the study lasted 4 weeks, with a 2-week washout period (during which the volunteers kept the low-salt diet). After reducing salt intake to below 100 mmol/day, participants were assigned to drink 1.5 L/day of: (a) a sodium chloride-rich^⊗ a^ mineral water (^#^ 56.3 mmol/L sodium, 42.5 mmol/L chloride, 14.4 mmol/L bicarbonate, 1.6 mmol/L calcium, 1.8 mmol/L potassium, 0.4 mmol/L magnesium and 2.9 mmol/L sulphate); (b) a sodium bicarbonate-rich^⊗ b^ mineral water (^#^ 26.2 mmol/L sodium, 4.3 mmol/L chloride, 32.5 mmol/L bicarbonate, 3.1 mmol/L calcium, 0.7 mmol/L potassium, 2.2 mmol/L magnesium and 0.7 mmol/L sulphate); and (c) a low-sodium mineral water (placebo: sodium, chloride, bicarbonate, calcium, potassium, magnesium and sulphate < 0.02 mmol/L). Dietary counselling was provided and compliance with treatments was checked. Determinations were done before the start of the study and at the end of the first^c^ and fourth weeks of each mineral water phase [^c^24 h ambulatory BP, circulating lipids and circulating glucose and insulin response to an oral glucose load (75 g) were not evaluated at this time point].	Versus baseline, mean resting BP, at weeks 1 and 4, was significantly reduced when consuming low-sodium or sodium bicarbonate-rich mineral waters but unchanged with sodium chloride-rich mineral water. At week 4, ambulatory 24 h BP was not altered by any of the 3 waters. At weeks 1 and 4, urinary sodium was significantly reduced by the ingestion of low-sodium mineral water versus baseline, significantly increased with sodium chloride-rich and sodium bicarbonate-rich mineral water ingestion versus low-sodium mineral water and significantly reduced with sodium bicarbonate-rich mineral water ingestion versus sodium chloride-rich mineral water (and non-significantly versus baseline); urinary chloride showed a pattern similar to sodium (although not always with the same statistical significance). At weeks 1 and 4, urinary calcium was significantly reduced by drinking low-sodium and sodium bicarbonate-rich mineral waters versus baseline (non-significantly for low-sodium mineral water at week 1); with sodium chloride-rich mineral water intake a non-significant decrease versus baseline was observed only at week 4. At weeks 1 and 4, urinary net acid increased with sodium chloride-rich (significantly at both time points) and low-sodium mineral water ingestion (significantly only at week 1) versus baseline. No effects were observed in circulating catecholamines and uric acid as well as in glomerular filtration rate under the 3 distinct mineral water treatments. Although circulating renin activity, aldosterone and atrial natriuretic factor values were distinctly affected by the 3 different interventions at end of week 1, at the end of week 4 there were no differences versus baseline.
	^⊗ a^ Water classified as mineral-rich, bicarbonate-rich, sodium-rich, chloride-rich and sulphate-rich. ^⊗ b^ Water classified as mineral-rich, bicarbonate-rich, sodium-rich and magnesiac.		
[65]	10 Patients, men (n = 4) and women (n = 6), aged 55–74 y [65 ± 6.7 y (mean ± SD)]; with moderate hypercholesterolemia (total-cholesterol > 240 mg/dL; LDL-cholesterol > 170 mg/dL). With exclusion criteria. Clinical sequential trial.	To evaluate the effect of a spring mineral water^⊗^ ingestion in moderate hypercholesterolemia (Table 2). The study was conducted over a 9-week period. The first 3 week-period matched the dietary stabilization phase (dietary-lead-in period) that was followed by 3 weeks of active treatment with mineral water and 3 further weeks of control treatment with tap water. During the study, the subjects were given a standard daily diet and, for each of the 2 treatments, consumed, respectively, 0.75 L of Montecatini Regina^®^ mineral water (1540 mg/L sulphate, 136 mg/L magnesium, 5535 mg/L sodium, 9220 mg/L chloride and 677 mg/L bicarbonate) ^###^ or tap water (no information provided regarding its composition), in the morning. Blood sample collection was performed after 13 h of overnight fasting. Circulating lipids, ApoAI and ApoB were evaluated at T-21 (screening), T0 and every week during the 2 treatment periods [similar for circulating Lp(a) and identical for BP (no information found for circulating electrolytes)]. Feces collection was done for 24 h periods. Fecal total bile acids were evaluated on the last 3 days of the dietary-lead-in period, every day during the active treatment (five-day mean data) and once a week during the control period (similar for fecal fractionated bile acids). Gallbladder volume was evaluated just before and after water intake (at 15, 30, 45 and 60 min).	BP remained stable during the entire study. Circulating chloride significantly increased during the active treatment period but did not show any differences between the dietary-lead-in and control periods; circulating sodium, potassium and magnesium did not change significantly during the active treatment period.
	^⊗^ Water classified as mineral-rich, bicarbonate-rich, sodium-rich, chloride-rich, sulphate-rich and magnesiac.		
[59]	70 Women and men, aged 45–64 y, with borderline hypertension (SBP 15 mmHg above normal values for corresponding age, DBP ˃ 90 mmHg and within 20% of ideal BW, and with low urinary magnesium and calcium levels) and living in an area with low magnesium content in the drinking water. With exclusion criteria. Randomized parallel double-blinded trial, including 3 groups.	To determine the effects of the consumption of mineral water^⊗^ and water with added magnesium in borderline hypertension, with low urinary magnesium and calcium levels. Subjects consumed at least 1 L/day, for 4 weeks. The participants were allocated in 3 groups: (a) water low in minerals (n = 18; Valvert^®^; 2 mg/L magnesium, 67.6 mg/L calcium, 1.9 mg/L sodium, 0.2 mg/L potassium, 18 mg/L sulphate, 204 mg/L bicarbonate, 4 mg/L chloride, < 0.05 mg/L fluoride and 5.7 mg/L silica); (b) magnesium-enriched water (n = 18; distilled water + magnesium sulphate; 82.3 mg/L magnesium, 4 mg/L calcium, 2.4 mg/L sodium, 0.1 mg/L potassium, 326 mg/L sulphate, 12 mg/L bicarbonate, 0.7 mg/L chloride and 0 mg/L fluoride and silica); and (c) water high in minerals (n = 19; Contrex^®^; 84 mg/L magnesium, 486 mg/L calcium, 9.1 mg/L sodium, 3.2 mg/L potassium, 1187 mg/L sulphate, 403 mg/L bicarbonate, 8.6 mg/L chloride, 0.32 mg/L fluoride and 8 mg/L silica). Dietary control was done; compliance with treatments was checked. For analysis, volunteers with serum or urine values higher than the 75th percentile (n = 15) were excluded (a group with a sufficient body burden of minerals and not influenced by the intervention). Determinations were done before and after intervention periods. Additionally, BP was also evaluated at week 2.	A significant decrease in SBP and DBP was found at the second and fourth weeks in the group consuming Contrex^®^. Consumption of magnesium-enriched water and Contrex^®^ significantly increased urinary magnesium, but did not change urinary calcium, at the end of the protocol. No significant effects of treatments on circulating magnesium were detected. None of the subjects changed their normal dietary habits during the trial.
	^⊗^ Water classified as mineral-rich, sulphate-rich, calcic and magnesiac.		
[60]	31 Participants, aged 50–79 y (mean 62 y), 62% women, no smokers and without disease. With exclusion criteria. Clinical trial, with randomization, non-blinded.	To detect groups at risk for high BP and to assess the possibility to intervene in these groups with mineral water^⊗^. The participants consumed 75 mL/day of mineral water (split in five doses, evenly distributed over the day and without specific relation to mealtimes), for 2 weeks. Content per total daily dose of mineral water: 3.1 mmol magnesium, ^#^ 0.65 mmol calcium and 0.02 mmol potassium. No data on volunteers’ dietary habits were gathered. Determinations were undertaken at baseline and after 14 days, with and without intervention with mineral water.	At baseline, without mineral water ingestion, although positive significant associations between urinary urea and urinary calcium, magnesium and potassium were observed, the same did not happen for urinary urea versus SBP or DBP. Consistent results for the changes over time without mineral water intervention were observed. At baseline, without mineral water ingestion, among individuals with high urinary urea, lower urinary magnesium was significantly related to higher SBP; the same did not happen for potassium or calcium. No relationship occurred in the low urinary urea group. Mineral water ingestion induced a small and almost significant increase in urinary magnesium and calcium. Only among volunteers with initial high urinary urea and low urinary magnesium, mineral water intervention strongly tended to decrease DBP ^##^ (in 4 out of 5 subjects).
	^⊗^ Water classified as magnesiac.		
[49]	5 Healthy women [aged 27–61 y ^#^; 41 y (mean)]. Clinical trial, non-blinded.	To assess the effects of the ingestion of a mineral water^⊗^ in healthy subjects. ^#^ Volunteers drank 1 L Magnesia^®^ mineral water (204 mg/L magnesium, 39.5 mg/L calcium, 1.3 mg/L potassium and 5.4 mg/L sodium) in 30 min, on the 1st day; tests were made at 0, 2, 6, 24 and 48 h. Then, only 1 glass of water was drunk at a time by the individuals for 4 weeks, with a daily total of 1–1.5 L. Tests (but urine volume) were repeated after 1, 2, 3 and 4 weeks. No data on volunteers’ dietary habits were collected.	Magnesia^®^ water consumption significantly increased circulating and urinary magnesium as well as urine volume, in a specific time-dependent manner. Circulating and urinary magnesium peaked at 6 h, slowly decreasing afterwards (until 48 h). While urinary magnesium peaked again at the end of the first week and then decreased until the end of the fourth week (but always presenting higher values than baseline), circulating magnesium returned to baseline values. Urine volume increased from 2 to 48 h. BP, HR, ECG and circulating calcium, potassium and sodium did not vary.
	^⊗^ Water classified as magnesiac.		

ApoAI, apolipoprotein AI; ApoB, apolipoprotein B; BMI, body mass index; BP, blood pressure; BW, body weight; DBP, diastolic blood pressure; ECG, electrocardiogram; HR, heart rate; LDL, low-density lipoprotein; Lp(a), lipoprotein(a); Ref, reference; SBP, systolic blood pressure; SD, standard deviation. ^#^ in the abstract and body of manuscript different value(s) is(are) given; ^##^ SBP in the article abstract; ^###^ only partial composition provided in the original article (units were adjusted); ^$^ although the effect is mentioned in the text, the postprandial blood pressure values are not given in the corresponding table; ^⊗^ water classification according to reference [47].

**Table 2:** The influence of mineral water consumption upon lipid profile.

Ref	Population and Study Type; Water Classification	Aim and Intervention	Results
Studies in animal models.			
[57]	Information provided in Table 1.	Information provided in Table 1.	Circulating total-triglycerides significantly increased in FRUCT versus CONT rats, but just tended to increase in FRUCTMIN versus CONT rats. Circulating total-, LDL- and HDL-cholesterol did not differ among the 3 groups neither did the HDL-cholesterol/total-cholesterol and HDL-cholesterol/LDL-cholesterol ratios.
[70]	100 Specific pathogen-free male CD rats (100 ± 10 g). Randomized intervention, including 3 groups in parallel.	To study the impact of hydropinic treatment with mineral water^⊗^ in hypercholesterolemia (Table 4). Dietary intervention lasted 4 months. Rats were assigned into 3 groups: (a) CC group: tap water (TW) + standard diet (n = 30); (b) TC group: TW + hypercholesterolemic diet (2% cholesterol + 2% cholic acid; n = 35); and (c) TT group: San Giovanni^®^ spring mineral water (IDCS water) + hypercholesterolemic diet (n = 35). IDCS water (rich in minerals) contained 485 mg/L calcium, 209 mg/L magnesium, 50.9 mg/L potassium, 1650 mg/L chloride, 793 mg/L sodium, 714 mg/L bicarbonate and 1287 mg/L sulphate. 1^−^ and F^−^content amounted to 24.8 and 1.6 mg/L, respectively, in IDCS but was absent in TW. TW (with low-mineral content) contained 49.8 mg/L calcium, 0.96 mg/L magnesium, 0.24 mg/L potassium, 3.2 mg/L chloride, 1.74 mg/L sodium, 152.9 mg/L bicarbonate and 2.14 mg/L sulphate. During the last night of the interventions, feces were collected. At the end of the treatment, after 18 h of overnight fasting, blood samples were collected, intestinal propulsion was evaluated and bile flow (and bile composition) after biliary drainage was determined.	TC and TT rats presented significantly higher circulating total-, HDL- and LDL-cholesterol than CC rats. TT rats showed significant reductions in circulating total- and LDL-cholesterol versus TC rats (no differences were found in circulating HDL-cholesterol and total-triglycerides). TT rats had total-cholesterol/HDL-cholesterol, total-cholesterol/LDL-cholesterol and HDL-cholesterol/LDL-cholesterol cardiovascular risk indexes intermediate between those found for CC and TC rats. Total fecal bile acid elimination was significantly higher in TT than in TC rats. Intestinal propulsion was significantly higher in TT than in TC rats. TT and TC groups displayed similar biliary elimination curves, with alike total bile acids and cholesterol content in the bile.
	^⊗^ Water classified as mineral-rich, bicarbonate-rich, sodium-rich, chloride-rich, sulphate-rich, sulphurous, calcic, magnesiac and fluorurate.		
Studies in humans.			
[61]	Information provided in Table 1.	Information provided in Table 1.	Circulating total-triglycerides, ApoAI and ApoB did not differ at the end of the 2 periods. Circulating total- and LDL-cholesterol significantly decreased as well as cardiovascular risk indexes (total-cholesterol/HDL-cholesterol and LDL-cholesterol/HDL-cholesterol ratios), while circulating HDL-cholesterol significantly increased, after CSBMW intake versus CLMW.
[58]	Information provided in Table 1.	Information provided in Table 1.	CSBMW versus CLMW consumption significantly reduced circulating total- and LDL-cholesterol as well as cardiovascular risk indexes (total-cholesterol/HDL-cholesterol and LDL-cholesterol/HDL-cholesterol ratios) and ApoB, at the eighth week. LDL-cholesterol/HDL-cholesterol ratio changed only between the fourth and the eighth week of CSBMW consumption. The other parameters showed a continuum reduction (ApoB not evaluated at the fourth week). Circulating HDL-cholesterol tended to increase with CSBMW versus CLMW intake, only at the eighth week. Circulating total-triglycerides and ApoAI as well as LDL-cholesterol/ApoB and HDL-cholesterol/ApoAI ratios remained stable with treatments.
[66]	Information provided in Table 1.	Information provided in Table 1.	No significant effects were observed with low-mineralized referent water both at fasting and postprandial states. No difference was observed between the 2 waters both at fasting and postprandial states. At fasting state, a significant decrease in circulating total- and VLDL-triglycerides and a tendency to a decrease in circulating VLDL-cholesterol was observed after SY water consumption (a similar non-significant pattern was noticed for referent water). At postprandial state, the results showed no significant differences either for circulating total- and VLDL-triglycerides or for circulating VLDL-, LDL- and HDL-cholesterol after SY water consumption.
[63]	Information provided in Table 1.	Information provided in Table 1.	With CLMW and CSBMW intake, and without significant differences between these groups, circulating total- and LDL-cholesterol as well as cardiovascular risk indexes (total-cholesterol/HDL-cholesterol, LDL-cholesterol/HDL-cholesterol and LDL-cholesterol/ApoB ratios) significantly decreased, while circulating ApoB significantly increased, with time; circulating oxidised LDL tended to decrease with time. Circulating HDL-cholesterol, total-triglycerides and ApoAI as well as HDL-cholesterol/ApoAI ratio remained constant throughout the trial.
[62]	Information provided in Table 1.	Information provided in Table 1.	Circulating total-triglycerides and total-, LDL- and HDL-cholesterol did not differ among the 3 different regimens (sodium-chloride-rich, sodium-bicarbonate-rich and low-sodium mineral water ingestion; results provided only for the fourth week).
[65]	Information provided in Table 1.	Information provided in Table 1.	Treatment with Montecatini Regina^®^ water significantly reduced circulating total- and LDL-cholesterol as well as ApoB and total-cholesterol/HDL-cholesterol ratio (versus the other 2 periods). No differences were found between dietary stabilization and control periods. Circulating HDL-cholesterol, total-triglycerides, ApoAI and Lp(a) did not significantly change during the active treatment period. The gallbladder volume was significantly reduced with Montecatini Regina^®^ water treatment (no significant effect with tap water treatment). Total fecal bile acids were significantly increased with Montecatini Regina^®^ water (versus the other 2 periods). The lithocholic/deoxycholic ratio increased from 1:9 to 3.8:6.2 with Montecatini Regina^®^ water.
[71]	19 Healthy subjects (7 men and 12 women), aged 26–59 y [46.9 ± 11.1 y (mean ± SD)]; BMI 23.0 ± 3.2 Kg/m^2^ (average ± SD). With exclusion criteria. Clinical sequential trial.	To elucidate the effects of mineral water^⊗^ consumption in healthy volunteers (Table 3). Tap water (TW) and bicarbonate-rich mineral water (BMW) sequential consumption periods lasted for a week each; the sequence was repeated twice. The participants drank 0.5 L/day of TW or BMW, divided into thrice daily (30–60 min before breakfast, lunch and dinner). BMW (from Nagayu hot spring) contained 2485 mg/L bicarbonate, 412 mg/L sodium, 182 mg/L chloride, 80 mg/L potassium, 291 mg/L magnesium, 177 mg/L calcium, 355 mg/L sulphate and 0.3 mg/L fluoride. TW (from Nishikawa water treatment plant) contained, respectively, 28, 10, 11, < 0.1, 1.9, 6.1, 6.9 and < 0.1 mg/L. For both waters, H_2_ S and HS^−^ < 0.1 mg/L. Participants were instructed to maintain their normal dietary habits during the test. Compliance with treatments was checked. Determinations were done on the first day of the test and last days of every week, after overnight fasting.	Apart from circulating calcium, BMW intake had no impact on circulating urate, total-, HDL- and LDL-cholesterol, total-triglycerides, sodium, chloride, magnesium and cortisol. A significant increase was reported for circulating calcium.
	^⊗^ Water classified as mineral-rich, bicarbonate-rich, sodium-rich, sulphate-rich, magnesiac and calcic.		
[69]	21 Adult men (n = 10) and women (n = 11), aged > * 18 and < 40 y [27.8 ± 4.5 y (mean ± SD)]; triglycerides < 2.82 mmol/L (250 mg/dL), moderately hypercholesterolaemic; BMI > 18 and < 30 Kg/m^2^ [23.8 ± 2.9 Kg/m^2^ (mean ± SD)]. With exclusion criteria. Randomized, cross-over, controlled trial, including 4 groups.	To investigate the effects of mineral water^⊗^ consumption, with or without a standard fat-rich meal, in moderately hypercholesterolaemia (Table 3). 4 Different treatments were applied: 0.5 L of control low-mineral water (CLMW) + standard fat-rich meal, 0.5 L of carbonic sodium-bicarbonate mineral water (CSBMW) + standard fat-rich meal, 0.5 L of CLMW and 0.5 L of CSBMW. CSBMW was rich in bicarbonate (2120 mg/L), sodium (1102 mg/L) and chloride (597 mg/L); its content in calcium, magnesium, potassium, sulphate and fluoride being 32.0, 9.4, 49.5, 45.3 and 0.9 mg/L, respectively; with carbon dioxide (3.9 g/L). CLMW contained 104, 8.7, 11, 33.4, 5.0, 2.0, 15.6 and < 0.2 mg/L, respectively; without carbon dioxide. Both mineral waters were from Vichy Catalán^®^. Volunteers attended the clinic 4 times at 1-week intervals, having followed instructions regarding dinner composition the evening before the study and having fasted overnight for at least 12 h; compliance with dinner instructions and absence of water intake in the previous 12 h were checked. Volunteers were allocated to a specific individual sequence of treatments (that included the 4 arms of the postprandial study) on the 1st study day. They were instructed not to deviate from their regular habits and to maintain their normal diet, body weight, alcohol consumption and exercise level. Blood samples were obtained at basal conditions and at postprandial times (30, 60 and 120 min).	Consumption of CSBMW without a standard fat-rich meal (versus CLMW) did not significantly affect any of the studied parameters. With the standard fat-rich meal, a time effect was observed with both waters for circulating total-triglycerides and CCK as well as gallbladder volume. Consumption of CSBMW + standard fat-rich meal induced a significantly lower increase in circulating total-triglycerides and CCK than intake of CLMW + standard fat-rich meal. Consumption of CSBMW + standard fat-rich meal significantly increased gallbladder volume versus CLMW + standard fat-rich meal. Gallbladder ejection fraction was significantly lower with CSBMW + standard fat-rich meal but AUC and peak contraction amplitude (lowest gallbladder volume) were significantly higher versus CLMW + standard fat-rich meal.
	^⊗^ Water classified as mineral-rich, bicarbonate-rich, sodium-rich and chloride-rich.		
[72]	69 Hyperlipidemic adults (circulating total-cholesterol and triglycerides > 200 mg/dL, LDL-cholesterol > 150 mg/dL, HDL-cholesterol < 40 mg/dL); aged 30–60 y. Randomized controlled trial, including 2 groups in parallel.	To assess the hypolipidemic effects of a mineral water^⊗^ intake in hyperlipidemic subjects. 32 Adults received 1 L/day of high-mineral content water for 1 month [1350 mg/L bicarbonate, 250 mg/L calcium, 48 mg/L magnesium, 150 mg/L sodium, 165 mg/L sulphate, 9 mg/L potassium]; and 37 adults received 1 L/day of low-mineral water for the same period (29 mg/L, 7 mg/L, 1.7 mg/L, 5 mg/L, 20 mg/L and 2 mg/L, respectively). No data on volunteers’ dietary habits were evaluated. At the end of treatment, changes in lipid profile were compared separately in each studied group or between groups.	Circulating total- and LDL-cholesterol decreased significantly in both intervention groups (with no significant differences between the 2 intervention groups). Circulating total-triglycerides and HDL-cholesterol did not vary significantly after both interventions.
	^⊗^ Water classified as mineral-rich, bicarbonate-rich and calcic.		
[68]	18 Healthy postmenopausal women (amenorrhoeic for at least 1 year), aged 51–59 y; BMI 26.89 ± 3.04 Kg/m^2^ (mean ± SD); no smokers. With exclusion criteria. Randomized, cross-over, controlled trial, including 3 groups.	To investigate the effects of drinking mineral water^⊗^, with a standard fat-rich meal, in healthy postmenopausal women. 3 Different treatments were applied: standard fat-rich meal + 0.5 L of 1 of the 3 mineral waters tested [sodium-bicarbonate mineral water (SBMW) 1, SBMW2 and control low-mineral water (CLMW)]. SBMW1^⊗^ and 2^⊗^ were rich in bicarbonate (2094.4/2013 mg/L), sodium (1116.5/948 mg/L) and chloride (583.0/592 mg/L); their content in calcium, magnesium, potassium, sulphate and fluoride being 43.6/52.1, 5.7/9.7 mg/L, 54.7/47.7, 49.9/42.9 and 7.9/1.4 mg/L, respectively. CLMW was low in minerals (71.1, 9.0, 5.7, 25.2, 2.7, 1.4, 15.7 and 0.2 mg/L, respectively). All waters were from Vichy Catalán^®^. Volunteers attended the laboratory facilities on 3 occasions at 2-week intervals, having followed instructions regarding dinner composition the evening before the study and having fasted overnight for at least 12 h; compliance with dinner instructions was verified with a questionnaire. On the first study day, volunteers were assigned to a specific individual treatments sequence including the 3 arms of the postprandial study. Volunteers were instructed not to deviate from their regular habits and to maintain their normal diet and body weight, alcohol consumption and exercise level. Blood samples were obtained at basal conditions and at postprandial times (30, 60, 120, 240, 360 and 420 min).	Over the period of postprandial evaluation, no significant timeXwater effect was observed for any of the 4 variables evaluated (and time to peak did not significantly vary among treatments). Circulating total- and chylomicron-triglycerides showed significant water and time effects. Circulating total- and chylomicron-cholesterol showed no water effect, while a significant time effect was observed only for the latter. Only peak concentration of circulating total-triglycerides showed a significant water effect (with both SBMW1 and 2 similarly lower versus CLMW, but no significant differences were observed among the 3 treatments). Circulating chylomicron-cholesterol showed a similar but less intense and non-significant overall pattern of variation (both for postprandial TAUC and peak concentration). A significant water effect was observed only in TAUC of circulating total- and chylomicron-triglycerides [circulating total-triglycerides significantly lower with SBMW2 intake versus CLMW (SBMW1 and 2 consumption behaving similarly); chylomicron-triglycerides similarly lower with SBMW1 and 2 ingestion versus CLMW, but with no significant differences among the 3 treatments].
	^⊗^ Both waters classified as mineral-rich, bicarbonate-rich, sodium-rich, chloride-rich and fluorurate.		
[73]	40 Postmenopausal women with functional dyspepsia and/or constipation (amenorrhoeic for at least 1 year) [aged 61.2 ± 1.8 to 64.0 ± 1.4 y; BMI 24.0 ± 0.6 to 24.9 ± 0.9 Kg/m^2,^ for CTRL and THW groups, respectively (mean ± SE)]. With exclusion criteria. Non-blinded controlled trial, including 2 groups.	To investigate the effect of drinking thermal mineral^⊗^ water (THW) in postmenopause (Table 4). Interventions lasted 12 d; 0.5 L of each water (THW and tap water) was ingested every day in the morning, in the fasted state. The THW group (n = 20) drank Acqua Santa of Chianciano Terme^®^, at a temperature of 33 °C (730 mg/L bicarbonate, 29.4 mg/L chloride, 41 mg/L sodium, 180 mg/L magnesium, 840 mg/L calcium, 7 mg/L potassium, 2 mg/L fluoride, 1840 mg/L sulphate; with 537 cc/L carbon dioxide). The control group (CTRL; n = 20) drank Rome tap water at a temperature of 10–12 °C (6.5 mg/L chloride, 5.5 mg/L sodium, 19 mg/L magnesium, 98 mg/L calcium, 3 mg/L potassium, 0.2 mg/L fluoride, 15 mg/L sulphate). On days 1 and 13, after an overnight fasting and before drinking water, procedures were done. Additionally, daily, body weight measurements and stool diary filling in were done. Daily diet was controlled, characterized and analysed.	No effects were observed within each group or between groups in circulating lipid profile (total-triglycerides, total-, HDL- and LDL-cholesterol). Gallbladder volume and circulating total bile acids did not significantly differ between THW and CTRL groups both at baseline and at the end of the study. Gallbladder volume was significantly smaller at the end of the study than at baseline in THW group but not in CTRL group while circulating total bile acids were higher. THW group with significantly higher number of daily bowel movements versus CTRL group.
	^⊗^ Water classified as mineral-rich, sulphate-rich, bicarbonate-rich, magnesiac, calcic and fluorurate.		

ApoAI, apolipoprotein AI; ApoB, apolipoprotein B; AUC, area under the curve; BMI, body mass index; CCK, cholecystokinin; CONT, CD Sprague–Dawley rats with access to tap water; FRUCT, CD Sprague–Dawley rats with access to 10% fructose in tap water; FRUCTMIN, CD Sprague–Dawley rats with access to 10% fructose in mineral water; HDL, high-density lipoprotein; LDL, low-density lipoprotein; Lp(a), lipoprotein(a); Ref, reference; SD, standard deviation; SE, standard error; SY, Saint-Yorre mineral water; TAUC, total area under the curve; VLDL, very low-density lipoprotein. * Some inaccuracy between the information provided in the abstract and body of the article (in the exclusion criteria); ^⊗^ water classification according to references [47] and ([74] for H_2_S).

**Table 3:** The influence of mineral water consumption upon glucose.

Ref	Population and Study Type; Water Classification	Aim and Intervention	Results
Studies in animal models.			
[57]	Information provided in Table 1.	Information provided in Table 1.	Circulating glucose seemed to increase in FRUCT versus CONT rats. Circulating insulin and leptin significantly increased in FRUCT versus CONT group. Insulin sensitivity index strongly tended to decrease in FRUCT versus CONT rats. Mineral water ingestion appeared to counteract all the above-mentioned fructose-induced effects.
[74]	42 Male Wistar rats with STZ-induced diabetes. Randomized intervention, with 6 groups in parallel.	To investigate the effects of mineral water^⊗^ on cardiac fibrosis in diabetic rats (Table 4). The intervention lasted 7 weeks; rats were divided into: (a) untreated-diabetic rats; (b) diabetic rats drinking mineral water from the Thermal Center of Helwan (Helwan Kabritage); (c) diabetic rats intraperitoneally injected with NaHS (14 micromol/kg/day); (d) control rats; (e) control rats drinking mineral water (as before); and (f) control rats injected with NaHS (as before). Mineral water composition: chloride 1560 mg/L, sodium 1300 mg/L, sulphate 844 mg/L, calcium 403 mg/L, bicarbonate 180 mg/L, magnesium 49 mg/L, fluoride 1.5 mg/L and potassium 2 mg/L (also with a sulfuric degree of 8.4 mg/L).	Overall, no differences were observed with control rats versus the 2 treatments groups: only circulating insulin significantly decreased in NaHS versus mineral water group. Diabetic rats had significantly higher circulating glucose and HbA1C than normal controls; the opposite for circulating insulin, C-peptide and IGF-I. Mineral water intake by or NaHS injection to diabetic rats significantly reduced circulating glucose and HbA1C versus diabetic rats; the opposite for circulating insulin, C-peptide and IGF-I.
	^⊗^ Mineral-rich water, chloride-rich, sodium-rich, sulphate-rich, sulphurous, calcic and fluorurate.		
Studies in humans.			
[61]	Information provided in Table 1.	Information provided in Table 1.	Circulating glucose significantly decreased in CSBMW versus CLMW intake.
[58]	Information provided in Table 1.	Information provided in Table 1.	Circulating glucose strongly tended to decrease with CSBMW versus CLMW intake, similarly at the fourth and eighth weeks. Circulating insulin remained constant with treatments.
[66]	Information provided in Table 1.	Information provided in Table 1.	Circulating glucose remained constant with treatments with referent and SY waters, both at fasting and postprandial states.
[63]	Information provided in Table 1.	Information provided in Table 1.	Circulating glucose significantly decreased with time, equally with CLMW and CSBMW ingestion. Circulating insulin remained constant throughout the trial.
[62]	Information provided in Table 1.	Information provided in Table 1.	Circulating glucose and insulin levels, as well as their AUC (in the oral glucose tolerance test), did not differ among the 3 different regimens (sodium-chloride-rich, sodium-bicarbonate-rich and low-sodium mineral water ingestion), at any of the time points evaluated.
[71]	Information provided in Table 2.	Information provided in Table 2.	Circulating glycoalbumin significantly decreased during BMW versus TW interventions. Circulating glucose^d^ and insulin as well as HOMAR were not different between TW and BMW interventions. ^d^In serum, unlike in plasma, glucose slightly tended to be lowered during BMW versus TW treatments.
[69]	Information provided in Table 2.	Information provided in Table 2.	Circulating insulin remained constant with the treatments without food. When waters were consumed with the standard fat-rich meal, circulating insulin was non-significantly lower with CSBWM versus CLMW intake (at all time points). Circulating glucose did not change with consumption of the waters alone or with the standard fat-rich meal. With the standard fat-rich meal, a time effect was observed with both waters for circulating insulin, but not for circulating glucose.
[75]	18 Healthy postmenopausal women (amenorrhoeic for at least 1 year), aged 51–59 y [55.5 ± 2.28 y (mean ± SD)]. BMI < 30 Kg/m^2^ [26.88 ± 3.04 Kg/m^2^ (mean ± SD)]; no smokers. With exclusion criteria. Randomized, cross-over, non-blinded controlled trial, including 3 groups.	To study the effects of mineral water^⊗^ intake, with a standard fat-rich meal in healthy postmenopause. 3 Different treatments were applied: standard fat-rich meal + 0.5 L of 1 of the 3 mineral waters tested [sodium-bicarbonate mineral water (SBMW) 1, SBMW2 and control low-mineral water (CLMW)]. SBMW1^⊗^ and 2^⊗^ were rich in bicarbonate (2094.4/2013 mg/L), sodium (1116.5/948 mg/L) and chloride (583.0/592 mg/L). Their content in calcium, magnesium, potassium, sulphate and fluoride was 43.6/52.1, 5.7/9.7, 54.7/47.7, 49.9/42.9 and 7.9/1.4 mg/L, respectively. CLMW was low in minerals (71.1, 9.0, 5.7, 25.2, 2.7, 1.4, 15.7 and 0.2 mg/L, respectively). Both waters were from Vichy Catalán^®^. Volunteers attended the laboratory facilities on 3 occasions at 2-week intervals, after 12 h of overnight fasting. Written instructions were provided regarding dinner composition the evening before the study and compliance with dinner instructions was checked. Volunteers were assigned to an individual sequence of treatments including all 3 waters. Volunteers were instructed not to deviate from their regular habits and to maintain their normal diet and exercise level. Blood samples were obtained at basal and at postprandial times (30, 60, 120 min).	Glycemia did not change along the postprandial period for any of the 3 waters tested. Postprandial circulating insulin and glucose peak concentrations as well as time to peak did not differ for all 3 waters. At 120 min, SBMW1 ingestion significantly lower postprandial circulating insulin versus CLMW (at this point, a similar non-significant pattern was found for SBMW2 versus CLMW but no difference was found between SBMW1 and SBMW2). At 30 min, it showed non-significant higher values with both SBMW1 and SBMW2 versus CLMW intake. Postprandial circulating insulin showed significant time for all 3 waters and waterxtime effects, depending on HOMA values: it showed significantly different responses to mineral water intake depending on HOMA values. It showed nearly significant different postprandial responses between HOMA n-tile 1 and 3. HOMA3 group showed higher postprandial circulating insulin at 30 min than in HOMA1 and 2 groups (particularly for SBMW1 intake). With SBMW1 ingestion, postprandial circulating glucose in HOMA3 group was also slightly higher versus SBMW2 and CLMW intake. HOMA and QUICKY were significantly correlated and equally valid to assess differences in glucose and insulin metabolism. All women presented an adequate insulin metabolism based on HOMA data.
	^⊗^ Both waters classified as mineral-rich, bicarbonate-rich, sodium-rich, chloride-rich and fluorurate.		

AUC, area under the curve; BMI, body mass index; BMW, bicarbonate-rich mineral water; CLMW, control low-mineral water; CONT, CD Sprague–Dawley rats with access to tap water; CSBMW, carbonic sodium-bicarbonate mineral water; FRUCT, CD Sprague–Dawley rats with access to 10% fructose in tap water; FRUCTMIN, CD Sprague–Dawley rats with access to 10% fructose in mineral water; HbA1C, glycated hemoglobin; HOMA (HOMAR), homeostasis model assessment index (ratio); IGF-I, insulin growth factor-1; NaHS, sodium hydrosulfide; QUICKI, quantitative insulin sensitivity check index; Ref, reference; SD, standard deviation; STZ, streptozotocin; SY, Saint-Yorre mineral water; TW, tap water; ^⊗^ water classification according to references [47] and ([74] for H_2_S).

**Table 4:** The influence of mineral water consumption upon waist circumference (and other obesity-related parameters).

Ref	Population and Study Type	Aim and Intervention	Results
Studies in animal models.			
[57]	Information provided in Table 1.	Information provided in Table 1.	Overall, on each separate week (week by week), body weight and food ingestion displayed similar values for all groups. Week by week, fluid ingestion was significantly higher in both fructose-fed groups versus CONT group, without any significant difference between FRUCT and FRUCTMIN groups. Week by week, total energy ingestion was significantly higher for both fructose-fed groups versus CONT group, without any significant difference between FRUCT and FRUCTMIN groups. With time (over the 8-week period), a significantly higher and similar body weight increase in FRUCT and FRUCTMIN versus CONT rats occurred. With time, FRUCTMIN rats decreased food ingestion significantly more than CONT rats and presented a trend towards a higher decrease than FRUCT rats. With time, fluid ingestion increased significantly for FRUCTMIN versus CONT and FRUCT rats. With time, total energy ingestion decreased similarly for all groups.
[70]	Information provided in Table 2.	Information provided in Table 2.	TT rats with statistically higher body weight increase than TC rats. TT and TC rats grew significantly less than CC rats.
[74]	Information provided in Table 3.	Information provided in Table 3.	Body weight of diabetic rats was significantly lower than of controls. Body weight of diabetic rats + mineral water was similar to controls and significantly higher than of diabetic rats (similarly for diabetic rats + NaHS, although more weak).
Studies in humans.			
[61]	Information provided in Table 1.	Information provided in Table 1.	Body weight and BMI remained constant during the study. WC did not differ between the 2 treatment periods. Dietary energy intake remained constant throughout the entire study. The protein, carbohydrate, fat (including lipid profile), cholesterol and fiber intakes did not differ between the 2 treatment periods.
[58]	Information provided in Table 1.	Information provided in Table 1.	Body weight, BMI and WC remained constant during the study. Dietary energy intake did not vary during the entire study. No changes in protein, carbohydrate, fat, cholesterol, plant phytosterol and fiber intakes, and the type of fat ingested did not differ between the 2 intervention periods.
[63]	Information provided in Table 1.	Information provided in Table 1.	BMI and WC did not vary throughout the trial. Men exhibited significantly higher WC than women. No significant timexwater effects were observed upon nutrients intake except for saturated fat (opposite trend in CSBMW versus CLMW group) and for sodium (CSBMW was a source of this nutrient). A time effect was noticed for energy and carbohydrates intakes from beverages, while lipids, protein, and alcohol intakes remained unchanged. Soft drinks and fruit juice intakes significantly decreased in both treatments, while total fluid intake significantly increased with both mineral waters.
[56]	Information provided in Table 1.	Information provided in Table 1.	None of the 2 treatments had any effect on body weight.
[64]	Information provided in Table 1.	Information provided in Table 1.	None of the 2 treatments had any effect on body weight and BMI. Normal dietary habits did not change during the trial.
[73]	Information provided in Table 2.	Information provided in Table 2.	Body weight was similar in THW and CTRL groups both at baseline and at the end of the study. Similarly, no change in body weight was observed at the end of the study versus baseline when each group was individually considered. The number of pasta, meat and vegetable portions consumed during the study was significantly higher (approximately 2-fold) but bread consumption was significantly lower (half the amount) in THW versus CTRL group. No intergroup difference was found with regard to fish, pizza, legume, fruit, dessert, soft drink, milk, dairy product and espresso coffee consumption.

BMI, body mass index; CLMW, control low-mineral water; CONT, CD Sprague–Dawley rats with access to tap water; CSBMW, carbonic sodium-bicarbonate mineral water; CTRL, women drinking Rome tap water; FRUCT, CD Sprague–Dawley rats with access to 10% fructose in tap water; FRUCTMIN, CD Sprague–Dawley rats with access to 10% fructose in mineral water; NaHS, sodium hydrosulfide; Ref, reference; TC, pathogen-free male CD rats drinking tap water and consuming a hypercholesterolemic diet; TT, pathogen-free male CD rats drinking mineral water and consuming a hypercholesterolemic diet; THW, women drinking thermal mineral water; WC, waist circumference.
